# Health Literacy Varies According to Different Background Disease Natures and Characteristics of Participants for Patient Support Groups

**DOI:** 10.3390/ijerph17165702

**Published:** 2020-08-07

**Authors:** Chieh-Liang Wu, Chia-Hua Liou, Shih-An Liu, Wayne H.-H. Sheu, Shang-Feng Tsai

**Affiliations:** 1 Department of Critical Care Medicine, Taichung Veterans General Hospital, Taichung 407, Taiwan; cljeff.wu@gmail.com; 2 Department of Automatic Control Engineering, Feng Chia University, Taichung 407, Taiwan; 3 Center for Quality Management, Taichung Veterans General Hospital, Taichung 407, Taiwan; chliou@vghtc.gov.tw (C.-H.L.); andyliu7@gmail.com (S.-A.L.); 4 School of Medicine, National Yang-Ming University, Taipei 112, Taiwan; 5 Department of Otolaryngolog, Taichung Veterans General Hospital, Taichung 407, Taiwan; 6 Division of Endocrinology and Metabolism, Department of Medicine, Taichung Veterans General Hospital, Taichung 407, Taiwan; whhsheu@vghtc.gov.tw; 7 Institute of Biomedical Sciences, National Chung Hsing University, Taichung 402, Taiwan; 8 School of Medicine, National Defense Medical Center, Taipei 114, Taiwan; 9 Division of Nephrology, Department of Internal Medicine, Taichung Veterans General Hospital, Taichung 407, Taiwan; 10 Department of Life Science, Tunghai University, Taichung 407, Taiwan

**Keywords:** mandarin multidimensional health literacy questionnaire (MMHLQ), health literacy, patient support group (PSG), disease nature, participant

## Abstract

Introduction: Patient support groups (PSGs) should be designed according to the backgrounds of participants and the nature of their diseases. Using health literacy as an outcome indicator for PSGs is rare. Methods: All questionnaires (Mandarin Multidimensional Health Literacy Questionnaire, MMHLQ) were collected from eight PSGs to evaluate the health literacy of participants. Background data of participants were also collected, including patient or family, age, and first-time participation or not. Results: A total of 458 questionnaires were collected from eight PSGs. The diseases were: autoimmune disease (systemic lupus nephritis (SLE), malignancy (head and neck cancer (HNC), chronic disease (diabetes mellitus or DM), chronic kidney disease (CKD), hemodialysis and chronic obstructive pulmonary disease (COPD), genetic disease (autosomal dominant polycystic kidney disease (ADPKD), and degenerative disease (osteoporosis). For vasculitis (42.21 ± 16.49 years old for SLE) and genetic disease (48.95 ± 17.58 years old for ADPKD), participants were younger. More significant differences between first-time participation and MMHLQ scores were found in disease of osteoporosis, CKD, COPD, and hemodialysis. More significant differences between role of participation (patients themselves or family) and MMHLQ scores were found in SLE, ADPKD, hemodialysis, and CKD. More significant differences between age (elderly or not) and MMHLQ score were found in HNC, DM, CKD, COPD, and osteoporosis. Conclusion: Background data of participants varied across different diseases. Different disease natures and patient background statuses should therefore demand different designs in PSG. MMHLQ before PSGs can be used to help to improve the PSG curriculum on the health literacy of patients.

## 1. Introduction

The outcome of patient care depends on correct diagnosis and timely treatment. According to the World Health Organization, patient care extends beyond the above factors and involves patients themselves. For example, supports from family members and peers also affect patient outcomes. Patients therefore need both health education and psychological support. A patient support group (PSG) is defined as “a group of people with common experiences and concerns who provide emotional and moral support for one another.” PSGs are known to be beneficial for nearly all diseases, including chronic disorder [1,2,3], genetic disease [4], malignancy [5,6,7], and degenerative disease [8], as well as for surgical patients [9,10,11]. For patients with diabetes mellitus (DM), PSG group education is more efficient and cost-effective than non-PSG group education [2]. Diabetes education in a group setting is equally effective in providing equivalent or slightly better improvements in glycemic control. As for genetic disease (e.g., Wilson disease [4] and autosomal dominant polycystic kidney disease (ADPKD) [12]), PSGs also improve patient outcomes. PSGs are also beneficial for patients undergoing surgery. Studies on post-bariatric surgery [9,10,11] reported that those patients in PSGs wanting a support group are significantly more likely to be struggling to control their weight. PSGs can help patients with weight loss maintenance, improving body image, and returning to work, which are all important parts of postoperative care. PSGs are known to be good for cancer patients in a number of aspects: educating patients/family, sharing the experience of illness, providing strength to patients, raising public awareness, and fundraising. A randomized outcome study of metastatic cancer patients showed that learning about other patients’ experiences improves coping mechanisms [13]. Despite the widely reported benefits of PSG, no study has yet been done to compare benefits among PSGs across different diseases.

Health literacy is the degree to which individuals have the capacity to obtain, process, and understand basic health information and services [14]. Low levels of health literacy are associated with poorer health outcomes and poorer use of healthcare services [15]. Therefore, multiple professional organizations recommend the usage of universal health literacy precautions to provide understandable and accessible information to all patients, regardless of their literacy or education levels [16]. Several versions of population-based or national-specific health literacy have been designed, such as the Rapid Estimate of Adult Literacy in Medicine (REALM) and the Test of Functional Health Literacy in Adults (TOFHLA) in America [14], the Health Literacy Questionnaire (HLQ) in Australia [17], the European Health Literacy Survey Questionnaire (HLS-EU-Q) in Europe [18], and All Aspects of Health Literacy Scale (AAHLS) in the United Kingdom [19]. The Mandarin Multidimensional Health Literacy Questionnaire (MMHLQ) was designed by Wei [20] to evaluate health literacy specifically for the mandarin-speaking population. It has five dimensions: access, understanding, appraising, and applying health information, and communication, and there are 20 self-reported items. Cross-validation analysis showed its good reliability [20]. In our previous study, we reported the use of MMHLQ in evaluating PSG outcome, which can improve the PSG curriculum. In the present study, we used MMHLQ to compare health literacy across PSGs of different diseases.

## 2. Subjects and Methods

### 2.1. Study Design and Population

In 2017, our hospital was the first to conduct a working group dedicated to the creation and improvement of PSGs. Every department was encouraged to set up a minimum of one PSG to hold a minimum of two activities a year. At the end, a total of 45 PSGs were set up in all (*n* = 25) departments in the hospital. The questionnaire (MMHLQ) created by Wei [20] was sent to participants of 8 PGSs (including all kinds of diseases) for evaluating patients’ health literacy while they were attending PSGs. These PSGs covered all kinds of disease nature: autoimmune disease (SLE) by rheumatology, malignancy (head and neck cancer or HNC by otorhinolaryngology), chronic disease (DM) by metabolism and endocrinology, chronic kidney disease (CKD) by nephrology, hemodialysis by nephrology and chronic obstructive pulmonary disease (COPD) by chest medicine), genetic disease (ADPKD by nephrology), and degenerative disease (osteoporosis by orthology). In each kind of disease nature, we chose more active PSGs for our study to obtain a higher response rate and avoid selection bias. The PSGs of SLE, HNC, DM, CKD, hemodialysis, COPD, ADPKD, and osteoporosis were ranked best or among the best in their category of disease in our institute according to previous records. The PSGs had a good stakeholder map, good participation rate by patients/family and staff, and good curriculum quality.

We collected basic background information of participants, including data such as age, first-time participation or not, and patient or family. Our study protocol was approved by the institute review board (approved number: No: CE20063A). Informed consent of patients and family was waived due to the pure data analysis nature of the study.

### 2.2. Outcome Analysis Based on Mandarin Multidimensional Health Literacy Questionnaire (MMHLQ)

The MMHLQ was designed by Wei et al. in 2017 for the mandarin-speaking population, to evaluate their 5 dimensions of health literacy (namely, access, understanding, appraising, and applying health information, and communication) [20]. There were 20 self-reported variables (supplementary data, Table S1), which included 4 questions/dimension. The score system was as follows: 4 points for “very easy”, 3 points for “easy”, 2 points for “difficult”, and 1 point for “very difficult”. According to the instructions of MMHLQ, the ability levels were: “inadequate” if score ≤ 2.5, “limited/problematic” if 2.5 < score ≤ 2.98, “sufficient” if 2.98 < score ≤ 3.52, and “excellent” if 3.52 < score ≤ 4. Questionnaires were sent to all participants (including patients and family) at the beginning of the PSG. We allowed 30 min for participants to complete the questionnaires before collecting the results. The author of MMHLQ, Professor Wei, approved the use of their MMHLQ for our study on 25 May 2018 (Table S2).

In addition to all scores of MMHLQ from all different kinds of PSGs, we also investigated the associations between all important information (age, first-time participation or not, patient or family participation, and the elderly or not) and different PSGs.

### 2.3. Statistical Analyses

Quantitative data were expressed as mean ± standard deviation. Categorical variables were compared using Chi-square likelihood ratio, and one-way ANOVA was used to compare means for distinct groups. The associations among the MMHLQs of all 8 PSGs and potential factors (firs-time participation or not, patient or family, and elderly or not) were also analyzed with the Student′s *t* test. A *p* value < 0.05 was considered of statistical significance. SPSS software (Statistical Package for the Social Science, version 20.0, Armonk, NY, USA) was used for statistical analyses.

## 3. Results

We collected a total of 458 completed questionnaires from the eight PSGs (for diseases: SLE, HNC, DM, ADPKD, hemodialysis, CKD, COPD, and osteoporosis). The return represented a 91.1% response rate (Table 1). Most participants (72.5%) were first-time participants in PSGs. Among them, those participants with relatively mild and chronic diseases showed more first-time participation: 97.6% for osteoporosis, 86.8% for CKD, and 80.2% for ADPKD. By contrast, those participants with systemic diseases or lethal diseases showed greater participation in PSGs (>1 time): 45.6% for SLE, 38.6% for DM, and 37.8% for COPD. Most participants (66.7%) for ADPKD were family members, while most participants for DM (77.1%), COPD (75.6%) and osteoporosis (72%) were patients themselves. We found younger participants for vasculitis (42.21 ± 16.49 years old for SLE) and genetic disease (48.95 ± 17.58 years old for ADPKD). However, participants were older for chronic diseases (60.11 ± 15.48 years old for DM, 66.38 ± 14.08 years old for COPD) and degenerative disease (62.60 ± 11.05 for osteoporosis).

The detailed results of all 20 questions of MMHLQ according to PSGs are shown in Supplementary Table S1. For all participants (Table 1), the overall scores of individual dimensions were: 2.84 ± 0.25 for access, 3.06 ± 0.46 for understanding, 2.66 ± 0.58 for appraisal, 2.79 ± 0.51 for application, and 2.89 ± 0.50 for communication. Generally speaking, the highest score was for the dimension of understanding (3.06 ± 0.46) and the lowest score was for the dimension of appraisal (2.66 ± 0.58). Participants with CKD, compared with the other seven PSGs, showed the highest scores in all five dimensions: access (12.13 ± 3.15), understanding (12.61 ± 2.41), appraisal (11.71 ± 3.07), application (12.39 ± 2.53) and communication (12.76 ± 2.32). Participants with HNC, compared with the other seven PSGs, showed the lowest scores in three dimensions: access (10.79 ± 1.76), understanding (11.79 ± 1.21), and application (10.47 ± 1.83). Participants with osteoporosis, compared with the other seven PSGs, showed the lowest scores in two dimensions: appraisal (9.85 ± 2.07) and communication (10.76 ± 1.96).

More significant differences were found between first-time participation and MMHLQ scores in patients with osteoporosis, CKD, COPD, and hemodialysis (scores of dimension vs. PSG divided by types of disease in Table 2) (detailed scores of all variables of PSGs divided by diseases and types of PSG are shown in Supplementary Tables S5 and S6, respectively). For osteoporosis, first-time participants had lower scores in the following items: “evaluate the difference or consistence of health information” (2.45 ± 0.61 vs. 3.00 ± 0.00, *p* < 0.001), “evaluate the reliability of medical information from network” (2.28 ± 0.66 vs. 3.00 ± 0.00, *p* < 0.001), all four variables in the dimension of application (10.51 ± 1.88 vs. 12.00 ± 0.00, *p* < 0.001), and “talk to doctors about the chosen” (2.55 ± 0.61 vs. 3.00 ± 0.00, *p* < 0.001). For COPD, first-time participants had higher scores in “find health information from network” (3.00 ± 0.61 vs. 2.47 ± 0.72, *p* = 0.017), and lower scores in “evaluate whether the health information can be used to solve medical problems” (2.75 ± 0.59 vs. 3.12 ± 0.49, *p* = 0.035) and “evaluate whether the health information is suitable for oneself or not” (2.75 ± 0.65 vs. 3.12 ± 0.33, *p* = 0.016). For hemodialysis, first-time participants had a lower score in “get information about health protection” (2.71 ± 0.56 vs. 3.05 ± 0.38, *p* = 0.030). For CKD, first-time participants had higher scores in “follow the instructions on the medical bag to take medication” (3.24 ± 0.56 vs. 3.00 ± 0.00, *p* = 0.018), “evaluate whether the health information can be used to solve medical problems” (3.06 ± 0.75 vs. 2.20 ± 0.45, *p* = 0.018), “apply health information to know the progress of disease” (3.18 ± 0.73 vs. 2.40 ± 0.55, *p* = 0.028), “apply health information to decide how to treat disease” (3.18 ± 0.64 vs. 2.40 ± 0.55, *p* = 0.013), and all four variables of the dimension of communication (13.18 ± 2.10 vs.10.00 ± 1.87, *p* = 0.003).

More significant differences were found between the role of participation (patients themselves or family) and MMHLQ scores in patients with SLE, ADPKD, hemodialysis, and CKD (scores of dimension vs. PSG divided by types of disease in Table 3) (detailed scores of all variables of PSGs divided by diseases and types of PSG are shown in Supplementary Tables S7 and S8, respectively). For SLE, patient participation had higher scores in “Search for information about disease” (3.00 ± 0.55 vs. 2.59 ± 0.67, *p* = 0.021), “apply health information to prevent disease” (2.94 ± 0.55 vs. 2.64 ± 0.49, *p* = 0.039), and “apply health information to decide how to treat disease” (2.97 ± 0.52 vs. 2.41 ± 0.59, *p* = 0.001). For ADPKD, patient participation had higher scores in “understand the medication bag instructions” (3.22 ± 0.70 vs. 2.93 ± 0.54, *p* = 0.039), all four variables in the dimension of understanding (13.04 ± 2.17 vs. 11.78 ± 1.91, *p* = 0.009), “evaluate whether the health information can be used to solve medical problems” (2.93 ± 0.73 vs. 2.61 ± 0.63, *p* = 0.047), “evaluate the difference or consistence of health information” (2.96 ± 0.76 vs. 2.65 ± 0.62, *p* = 0.049), “apply health information to understand the report of health examination” (3.11 ± 0.64 vs. 2.78 ± 0.54, *p* = 0.016), and “ask medical personnel if you are not sure” (3.22 ± 0.51 vs. 2.83 ± 0.57, *p* = 0.004). For hemodialysis, patient participation had lower scores in “get information about health protection” (2.76 ± 0.52 vs. 3.06 ± 0.42, *p* = 0.046), and subtotal scores in the dimension of access (10.80 ± 1.63 vs. 11.89 ± 1.45, *p* = 0.029). For CKD, patient participation had lower scores in all four variables in the dimension of access (10.85 ± 3.45 vs. 13.56 ± 2.04, *p* = 0.006), “understand the instructions of medical personnel” (2.90 ± 0.72 vs. 3.39 ± 0.50, *p* = 0.021), “follow the instructions on the medical bag to take medication” (11.85 ± 2.41 vs. 13.44 ± 2.18, *p* = 0.040), all four variables in the dimensions of appraisal (10.45 ± 2.91 vs. 13.11 ± 2.68, *p* = 0.006), “apply health information to know the progress of disease” (2.85 ± 0.81 vs. 3.33 ± 0.59, *p* = 0.045) “apply health information to prevent disease“ (2.85 ± 0.67 vs. 3.33 ± 0.59, *p* = 0.25), and “apply health information to understand the report of health examination“ (2.95 ± 0.60 vs. 3.39 ± 0.50, *p* = 0.021).

More significant differences were found between age (elderly or not) and MMHLQ scores in patients with HNC, DM, CKD, COPD, and osteoporosis (scores of dimension vs. PSG divided by types of disease in Table 4) (detailed scores of all variables of PSGs divided by diseases and types of PSG are shown in Supplementary Tables S9 and S10, respectively). For HNC, the elderly had lower scores in “talk to doctors about the chosen” (2.83 ± 0.51 vs. 2.29 ± 0.49, *p* = 0.012). For DM, the elderly had lower scores in all four variables in the dimension of access (12.18 ± 2.21 vs. 10.35 ± 2.70, *p* = 0.003), “understand the medication bag instructions” (3.13 ± 0.47 vs. 2.87 ± 0.56, *p* = 0.041), “obey the instruction of medical personnel to care disease” (3.15 ± 0.49 vs. 2.87 ± 0.67, *p* = 0.045), “understand the instructions of medical personnel” (3.21 ± 0.41 vs. 2.81 ± 0.65, *p* = 0.003), “evaluate whether the health information can be used to solve medical problems” (2.95 ± 0.56 vs. 2.58 ± 0.62, *p* = 0.012), “evaluate the health information suitable for himself/herself or not” (2.90 ± 0.60 vs. 2.55 ± 0.68, *p* = 0.025), and in all four variables in the dimension of application (12.00 ± 1.10 vs. 10.61 ± 2.20, *p* = 0.003). For CKD, the elderly had lower scores in “search for information about disease” (3.21 ± 0.74 vs. 2.30 ± 0.82, *p* = 0.002), “get information about health protection” (3.21 ± 0.74 vs. 2.50 ± 0.71, *p* = 0.012), “find health information from network” (3.25 ± 0.80 vs. 2.40 ± 0.70, *p* = 0.005), “understand the medication bag instructions” (3.29 ± 0.60 vs. 2.70 ± 0.67, *p* = 0.015), “understand the instructions of medical personnel” (3.29 ± 0.60 vs. 2.70 ± 0.67, *p* = 0.015), “follow the instructions on the medical bag to take medication” (3.32 ± 0.55 vs. 2.90 ± 0.32, *p* = 0.007), in all four variables in the dimension of evaluation (12.50 ± 3.05 vs. 9.50 ± 1.90, *p* = 0.006), “apply health information to know the progress of disease” (3.29 ± 0.66 vs. 2.50 ± 0.71, *p* = 0.003), “apply health information to prevent disease” (3.21 ± 0.69 vs. 2.70 ± 0.48, *p* = 0.036), and “apply health information to understand the report of health examination” (3.29 ± 0.60 vs. 2.80 ± 0.42, *p* = 0.024). For COPD, the elderly had lower scores in “search for information about disease” (2.94 ± 0.64 vs. 2.52 ± 0.64, *p* = 0.035), “find health information from network” (3.11 ± 0.68 vs. 2.59 ± 0.64, *p* = 0.012), “get information about report of health examination report” (3.11 ± 0.68 vs. 2.63 ± 0.56, *p* = 0.013), “obey the instruction of medical personnel to care disease” (3.39 ± 0.50 vs. 2.96 ± 0.34, *p* = 0.004), “understand the instructions of medical personnel” (3.44 ± 0.51 vs 3.04 ± 0.34, *p* = 0.006), and “apply health information to prevent disease” (3.17 ± 0.38 vs. 2.78 ± 0.64, *p* = 0.026). For osteoporosis, the elderly had lower scores in “find health information from network” (3.05 ± 0.51 vs. 2.58 ± 0.82, *p* = 0.002) and “apply health information to understand the report of health examination” (2.90 ± 0.60 vs. 2.58 ± 0.54, *p* = 0.014).

## 4. Discussion

The background data of participants (including age, first-time or not participation, and patient or family) varied according to differences in the nature of the disease. First, first-time participation or not depends strongly on the nature of different PSGs. For those participants with mild, chronic disease (including degenerative disease), they tended to pay less attention because of the less acute illness and no insight into the disease. Therefore, most of our participants were first-time participants. By contrast, patients of severe diseases with greater comorbidity (such as systemic disease, including SLE, DM) or of lethal (HNC) diseases were more willing to attend PSGs. Second, regarding the role of participants, family members of patients with genetic diseases also worried about themselves due to the nature of inheritance. Therefore, for ADPKD, more participants were family members (66.7%), a proportion that was higher compared with those of other PSGs. Third, the ages of participants were also related to the nature of PSG. Patients with SLE or genetic diseases were younger due to the disease’s nature, whereas for malignancy, chronic, and degenerative disease, patients were older. This finding would be helpful in planning PSGs and adjusting the curricula. Detailed scores of MMHLQ divided by PSGs are shown in Supplementary Table S3. The MMHLQ and background data from different PSGs are also shown in Supplementary Table S4.

In all eight PSGs, the best ability was found in the dimension of understanding, and the worse in the dimension of appraisal. The dimension of appraisal is most important because of information explosion in the current era of data deluge [21]. Even for professional doctors, there were still some problems in dealing with more and more published medical journals at nearly exponential rates [21]. In recent years, we professional doctors have started to be taught how to appraise critically medical literature. Moreover, the problem of appraisal is more severe for lay persons. For all PSGs, we should provide the best practices to ensure healthcare consumers can better understand how to seek and obtain credible health information. However, there is still a difference in this among different PSGs. Comparing across the either PSGs, participants of CKD obtained the highest scores in nearly all five dimensions. Participants of HNC, on the other hand, had the lowest scores in three dimensions (access, understanding, and application), and participants of osteoporosis had the lowest scores in two dimensions (appraisal and communication). The differences were related to differences in the disease’s nature and in the healthcare environment. First, in Taiwan, the incidence of CKD [22] and the prevalence of end-stage renal disease [23] are both much higher compared with those of other countries. Renal disease is a major financial burden for the healthcare system. Therefore, the National health Insurance Administration Ministry of Health and Welfare in Taiwan has launched an integrated kidney disease care program [24,25], including education for patients. Thus, Taiwanese participants had good health literacy regarding CKD. By contrast, patients did not pay much attention to osteoporosis. Moreover, most patients believed that osteoporosis is a process associated with aging rather a disease [26]. In addition, few Taiwanese specialists have focused on osteoporosis to date. Older patients also had weaker cognitive and physical functions, which contributed to a poor health literacy. The three factors above likely led to poor health literacy in the osteoporosis PSG. Third, the nature of HNC was as follows: non-specific symptoms, difficulty in knowing the cause, hard-to-realize treatment plan, and complicated disease course. All of these contributed to poor health literacy. In summary, these different disease natures and patient background statuses should demand different PSG designs.

Regarding first-time participation, first-time participation in the PSG of osteoporosis was associated with low MMHLQ scores with statistical significance. One can design PSG curricula to compensate for poor health literacy. However, for CKD, firs-time participation was associated with higher scores of MMHLQ. First, that may be due to few the (only five) participants in this PSG having repeated participation. Second, there was some bias in this self-reported study [27], including fixed-choice questions that lacked flexibility, social desirability bias, acquiescence, set response, and a lower reliability. Self-reported answers may be exaggerated. According to Chao et al., self-reported CKD is not a good surrogate for laboratory-diagnosed CKD [28]. Additionally, the prevalence of self-reported cases was extremely low (only 2.1%) from a recent study [29]. These suggested a substantial bias in self-reporting data. Therefore, one should be cautious in interpreting findings from such self-reported data on health literacy. Those first-time participants, though already securing good health literacy, may also be too conceited or complacent. In the case of illusion, the disease outcome may be worse than unawareness or no insight. This result echoes those of other PSGs—e.g., CKD, whose participants obtained the highest scores in nearly all five dimensions. For this kind of PSG, one should ensure (such as with pre-test) that those people have adequate health literacy to avoid poor outcome due to self-overestimation.

The role of patients or family participation also mattered. For the PSGs of SLE and ADPKD, patients’ self-participation was associated with higher scores in MMHLQ. However, for the PSGs of HD and CKD, family predications were associated with higher scores in MMHLQ. This could be due to the fact that for renal disease, patients undergoing hemodialysis do not die as quickly as those with malignancy or other acute diseases. They often suffer from comorbidities or complications of hemodialysis for years. Families have to look after patients and also keep them company during hemodialysis for three times a week for long periods of time. Even without diseases, family members of hemodialysis patients may worry more about hemodialysis than the patients themselves. The relationship between patients undergoing hemodialysis and their family is known to be difficult [30]. Further, family members′ experiences matter [31]. Therefore, they may have better health literacy than patients themselves. This implies that education should be conducted not only for patients but for family on domestic healthcare. For this type of disease and PSG, one should initiate more family-centered care, tailor-made education, and interventions focusing on the care-providing partner [31].

The group of the elderly increased rapidly all over the world. According to the results of a German cross-sectional study [32], 66.3% of participants aged 65 years and above had limited health literacy. Furthermore, the severity of limited health literacy was much more significant among respondents above 76 years of age (80.6%) [32]. Another cross-sectional study in Taiwan also showed that health literacy was significantly negatively related to age (95% confidence interval (CI) = (−0.07; 0.00); *p* = 0.03) [33]. A Korean study in 2017 also had a similar result [34]. The results of the above studies were consistent to those of our study in that elderly people were associated with overall lower MMHLQ scores across all PSGs. However, our study is the first one to investigate the role of age in health literacy among PSGs of different diseases. In our study, this decreased health literacy is less marked in the PSGs of HNC, SLE, and ADPKD (42.21 ± 16.49, 53.40 ± 13.37, and 48.95 ± 17.58 years old, respectively). First, this may be because of the younger ages of participants in these three PSGs. By contrast, for chronic disease (e.g., DM, CKD, COPD and osteoporosis), the elderly group was strongly associated with poorer health literacy. Second, and more importantly, these three diseases typically manifest early in life, and therefore, patients have been informed earlier. With time, they gain better health literacy about their diseases. Therefore, by the time they reach an age at which they are considered elderly (65 years old), they have good health literacy still without any visual, auditory and cogitative impairments. If they live even longer, though, it is likely that their health literacy will also decline.

There are some limitations to our present study. Not all questionnaires for all 45 PSGs were obtained in our hospital. However, we had already collected up to 458 for all types of diseases as described above. We thus believe that our results can be extended to other PSGs. Second, we did not collect MMHLQ results before and after attending PSGs.

## 5. Conclusions

Participants’ background data (including age, first-time or not participation, and patient or family) were variable due to differences in the diseases’ nature. Different disease natures and patient background statuses should demand different PSG designs. MMHLQ before a PSG can be used to help to improve both PSG curriculum design and patients’ health literacy.

## Figures and Tables

**Table 1:** Background characteristics of participants and score of the Mandarin Multidimensional Health Literacy Questionnaire (MMHLQ) divided by patient support groups (PSGs) (*n* = 458) (mean ± SD).

Variable	SLE	Head-and-Neck Cancer	DM	ADPKD	Hemodialysis	CKD	COPD	Osteoporosis	Total	*p* Value
Case (*n*)	56	43	70	81	43	38	45	82	458	
Response rate (%)	93.3%	86.0%	87.5%	90.0%	86.0%	84.4%	90.0%	91.1%	91.1%	
1st time participation (*n*, %)	31	31	43	65	21	33	28	80	332	<0.001
	55.4%	72.1%	61.4%	80.2%	48.8%	86.8%	62.2%	97.6%	72.5%	
Patient (*n*, %), not family	34	27	54	27	25	20	34	59	280	<0.001
	60.7%	62.8%	77.1%	33.3%	58.1%	52.6%	75.6%	72.0%	61.1%	
Age (years old)	42.21	53.40	60.11	48.95	54.12	53.89	66.38	62.60	55.30	<0.001
	±16.49	±13.37	±15.48	±17.58	±13.07	±15.20	±14.08	±11.05	±16.39	
MMHLQ: 5 subscales										
Subscale 1: Access										
1. Search for information about disease	2.84	2.56	2.84	2.86	2.81	2.97	2.69	2.72	2.79	0.102
	±0.63	±0.63	±0.73	±0.63	±0.50	±0.85	±0.67	±0.63	±0.66	
2. Get information about health protection	2.89	2.77	2.90	2.95	2.88	3.03	2.93	2.84	2.90	0.616
	±0.56	±0.43	±0.66	±0.52	±0.50	±0.79	±0.65	±0.62	±0.60	
3. Find health information from network	3.05	2.79	2.76	2.94	2.84	3.03	2.80	2.80	2.87	0.194
	±0.62	±0.60	±0.81	±0.64	±0.69	±0.85	±0.69	±0.73	±0.71	
4. Get information about report of health examination report	2.71	2.67	2.87	2.91	2.72	3.11	2.82	2.72	2.81	0.014
	±0.53	±0.57	±0.64	±0.60	±0.55	±0.80	±0.65	±0.61	0.62	
Total of subscale	11.50	10.79	11.37	11.67	11.26	12.13	11.24	11.09	11.37	0.161
	±1.82	±1.86	±2.59	±2.17	±1.63	±3.15	±2.20	±2.14	±2.23	
Subscale 2: Understanding										
5. Understand the medication bag instructions	3.00	2.95	3.01	3.02	3.02	3.13	3.09	2.95	3.02	0.764
	±0.60	±0.43	±0.52	±0.61	±0.41	±0.66	±0.60	±0.54	±0.56	
6. Obey medical personnel instructions to care for disease	3.09	2.86	3.03	3.04	3.00	3.13	3.13	3.06	3.04	0.412
	±0.67	±0.52	±0.59	±0.58	±0.53	±0.70	±0.46	±0.51	±0.57	
7. Understand the instructions of medical personnel	3.13	2.91	3.03	3.05	3.05	3.13	3.20	3.07	3.07	0.239
	±0.47	±0.43	±0.56	±0.52	±0.43	±0.66	±0.46	±0.49	±0.51	
8. Follow the instructions on the medical bag to take medication	3.14	3.07	3.06	3.09	3.16	3.21	3.20	3.11	3.12	0.796
	±0.70	±0.34	±0.59	±0.57	±0.43	±0.53	±0.50	±0.59	±0.55	
Total of subscale	12.36	11.79	12.13	12.20	12.23	12.61	12.62	12.20	12.25	0.488
	±1.92	±1.21	±2.01	±2.08	±1.44	±2.41	±1.71	±1.69	±1.85	
Subscale 3: Appraisal										
9. Evaluate whether the health information can be used to solve medical problems	2.71	2.58	2.79	2.72	2.70	2.95	2.89	2.54	2.72	0.011
	±0.56	±0.50	±0.61	±0.68	±0.60	±0.77	±0.57	±0.63	±0.63	
10. Evaluate whether the health information is suitable for oneself or not	2.66	2.60	2.74	2.78	2.74	3.03	2.89	2.56	2.73	0.005
	±0.61	±0.54	±0.65	±0.61	±0.66	±0.72	±0.57	±0.59	±0.63	
11. Evaluate the difference or consistence of health information	2.45	2.65	2.70	2.75	2.63	2.89	2.87	2.46	2.66	0.001
	±0.63	±0.53	±0.69	±0.68	±0.62	±0.83	±0.63	±0.61	±0.67	
12. Evaluate the reliability of medical information from network	2.48	2.60	2.50	2.69	2.58	2.84	2.67	2.29	2.56	0.002
	±0.66	±0.62	±0.74	±0.68	±0.73	±0.89	±0.67	±0.66	±0.71	
Total of subscale	10.30	10.44	10.73	10.94	10.65	11.71	11.31	9.85	10.66	0.001
	±2.04	±1.84	±2.40	±2.40	±2.26	±3.07	±2.20	±2.07	±2.33	
Subscale 4: Application										
13. Apply health information to know the progress of disease	2.68	2.63	2.81	2.78	2.74	3.08	2.80	2.56	2.74	0.002
	±0.61	±0.62	±0.55	±0.63	±0.54	±0.75	±0.63	±0.52	±0.61	
14. Apply health information to prevent disease	2.82	2.63	2.83	2.83	2.84	3.08	2.93	2.66	2.81	0.004
	±0.54	±0.54	±0.54	±0.63	±0.37	±0.67	±0.58	±0.57	±0.57	
15. Apply health information to understand the report of health examination	2.84	2.70	2.90	2.89	2.93	3.16	2.80	2.73	2.86	0.004
	±0.56	±0.56	±0.46	±0.59	±0.40	±0.59	±0.59	±0.59	±0.56	
16. Apply health information to decide how to treat disease	2.75	2.51	2.84	2.77	2.79	3.08	2.82	2.60	2.76	<0.001
	±0.61	±0.55	±0.53	±0.62	±0.47	±0.67	±0.61	±0.54	±0.59	
Total of subscale	11.09	10.47	11.39	11.26	11.30	12.39	11.36	10.55	11.16	<0.001
	±1.84	±1.83	±1.80	±2.33	±1.34	±2.53	±2.17	±1.87	±2.04	
Subscale 5: Communication										
17. Talk to doctors about the chosen	2.86	2.74	2.80	2.94	2.81	3.21	2.91	2.56	2.83	<0.001
	±0.67	±0.54	±0.58	±0.56	±0.45	±0.62	±0.56	±0.61	±0.60	
18. Confirm with personnel the accuracy of orders	2.96	2.77	2.86	2.90	2.93	3.24	2.93	2.73	2.89	0.001
	±0.47	±0.68	±0.49	±0.58	±0.46	±0.54	±0.50	±0.59	±0.56	
19. Discuss with doctor about the choice of treatment	3.05	2.86	2.87	2.93	2.88	3.11	3.11	2.72	2.92	0.001
	±0.52	±0.60	±0.51	±0.61	±0.50	±0.69	±0.49	±0.57	±0.57	
20. Ask medical personnel if you are not sure	2.98	2.84	2.87	2.96	2.95	3.21	3.04	2.74	2.93	0.001
	±0.36	±0.57	±0.54	±0.58	±0.43	±0.58	±0.56	±0.58	±0.55	
Total of subscale	11.86	11.21	11.40	11.73	11.58	12.76	12.00	10.76	11.57	<0.001
	±1.69	±2.05	±1.82	±2.15	±1.53	±2.32	±1.80	±1.96	±1.99	

Chi-square test. One-way ANOVA. MMHLQ: Mandarin Multidimensional Health Literacy Questionnaire, PSG: patient support group, SLE: systemic Lupus Erythematosus, DM: diabetes mellitus, ADPKD: autosomal dominant polycystic kidney disease, CKD: chronic kidney disease, COPD: chronic obstructive pulmonary disease.

**Table 2:** The association between first-time participation and all dimensions of MMHLQ divided by types of PSGs.

	Autoimmune Disease			Malignancy			Chronic Disease			Genetic Disease			Degenerative Disease		
	1st Time	Not 1st Time	*p* Value	1st Time	Not 1st Time	*p* Value	1st Time	Not 1st Time	*p* Value	1st Time	Not 1st Time	*p* Value	1st Time	Not 1st Time	*p* Value
Access	11.52	11.48	0.942	11.19	9.75	0.086	11.42	11.30	0.849	11.58	12.00	0.497	11.03	13.50	0.106
	±1.77	±1.92		±1.35	±2.53		±2.77	±2.32		±2.30	±1.59		±2.09	±3.54	
Understanding	12.26	12.48	0.671	12.00	11.25	0.117	12.07	12.22	0.759	12.18	12.25	0.911	12.18	13.00	0.498
	±1.95	±1.92		±1.06	±1.42		±2.19	±1.72		±2.10	±2.05		±1.67	±2.83	
Evaluation	10.26	10.36	0.854	10.68	9.83	0.542	10.84	10.56	0.635	10.83	11.38	0.420	9.83	11.00	0.432
	±2.18	±1.83		±1.68	±2.17		±2.39	±2.44		±2.50	±1.96		±2.09	±1.41	
Application	11.26	10.88	0.450	10.81	9.58	0.115	11.33	11.48	0.728	11.25	11.31	0.919	10.51	12.00	<0.001
	±1.61	±2.11		±1.49	±2.35		±1.98	±1.50		±2.47	±1.70		±1.88	±0.00	
Communication	11.84	11.88	0.929	11.48	10.50	0.161	11.26	11.63	0.407	11.68	11.94	0.667	10.70	13.00	0.101
	±1.71	±1.69		±2.01	±2.07		±1.83	±1.82		±2.21	±1.95		±1.94	±1.41	

Student’s *t*-test. MMHLQ: Mandarin Multidimensional Health Literacy Questionnaire, PSG: patient support group.

**Table 3:** The association between participation (patient or family) and all dimensions of MMHLQ divided by types of PSGs.

	Autoimmune Disease			Malignancy			Chronic Disease			Genetic Disease			Degenerative Disease		
	Not Family	Family	*p* Value	Not Family	Family	*p* Value	Not Family	Family	*p* Value	Not Family	Family	*p* Value	Not Family	Family	*p* Value
Access	11.82	11.00	0.098	10.74	10.88	0.822	11.44	11.13	0.668	12.19	11.41	0.130	10.98	11.35	0.491
	±1.87	±1.66		±1.95	±1.75		±2.63	±2.50		±2.08	±2.19		±2.25	±1.85	
Understanding	12.56	12.05	0.333	12.00	11.44	0.141	12.19	11.94	0.550	13.04	11.78	0.009	12.29	11.96	0.428
	±2.19	±1.40		±1.04	±1.41		±2.21	±1.12		±2.17	±1.91		±1.58	±1.97	
Evaluation	10.71	9.68	0.065	10.70	10.00	1.86	10.74	10.69	0.938	11.70	10.56	0.042	9.78	10.04	0.608
	±2.07	±1.86		±1.81	±1.86		±2.44	±2.30		±2.69	±2.17		±2.11	±2.01	
Application	11.62	10.27	0.006	10.63	10.19	0.451	11.33	11.56	0.659	12.00	10.89	0.042	10.39	10.96	0.221
	±1.83	±1.58		±1.80	±1.91		±1.85	±1.67		±2.72	±2.03		±1.91	±1.74	
Communication	12.06	11.55	0.270	11.52	10.69	0.203	11.41	11.38	0.951	12.41	11.39	0.044	10.75	10.78	0.940
	±1.84	±1.41		±1.93	±2.21		±1.96	±1.31		±2.22	±2.05		±1.94	±2.04	

Student’s *t*-test. MMHLQ: Mandarin Multidimensional Health Literacy Questionnaire, PSG: patient support group.

**Table 4:** The association between age (<65 or ≥65 years old) and all dimensions of MMHLQ divided by types of PSGs.

	Autoimmune Disease			Malignancy			Chronic Disease			Genetic Disease			Degenerative Disease		
	Age < 65	Age ≥ 65	*p* Valuae	Age < 65	Age ≥ 65	*p* Value	Age < 65	Age ≥ 65	*p* Value	Age < 65	Age ≥ 65	*p* Value	Age < 65	Age ≥ 65	*p* Value
Access	11.63	10.57	0.150	10.89	10.29	0.439	12.18	10.35	0.003	11.77	12.25	0.526	11.64	10.58	0.024
	±1.84	±1.40		±1.89	±1.70		±2.21	±2.70		±1.91	±3.07		±1.90	±2.24	
Understanding	12.41	12.00	0.603	11.92	11.14	0.122	12.64	11.48	0.015	12.34	11.63	0.362	12.13	12.26	0.735
	±1.91	±2.08		±1.20	±1.07		±1.60	±2.29		±1.81	±2.92		±1.88	±1.51	
Evaluation	10.31	10.29	0.975	10.56	9.86	0.365	11.28	10.03	0.029	10.94	10.94	0.999	10.08	9.65	0.356
	±2.11	1.50		±1.87	±1.68		±2.18	±2.51		2.19	±3.21		±2.25	±1.90	
Application	11.16	10.57	0.431	10.67	9.43	0.102	12.00	10.61	0.003	11.32	11.00	0.721	10.90	10.23	0.109
	±1.89	±1.51		±1.76	±1.99		±1.10	±2.20		±2.00	±3.43		±1.96	±1.76	
Communication	11.92	11.43	0.478	11.42	10.14	0.135	11.92	10.74	0.010	11.86	11.19	0.355	10.90	10.63	0.537
	±1.72	±1.51		±2.06	±1.77		±1.29	±2.18		2.01	2.66		±1.86	±2.06	

Student’s *t*-test. MMHLQ: Mandarin Multidimensional Health Literacy Questionnaire, PSG: patient support group.

## Supplementary Materials

The following are available online at https://www.mdpi.com/1660-4601/17/16/5702/s1, Table S1: Questionnaire of MMHLQ. Table S2. Certification for the approval by authors (Mi-Hsiu Wei et al.) for analysis on 25 May 2018. Table S3. Detailed score of MMHLQ divided by PSGs. Table S4. Background characteristics of participants and score of MMHLQ divided by types of PSGs. Table S5 The association between first time participation and all scores of MMHLQ divided by PSGs. Table S6. The association between first time participation and all scores of MMHLQ divided by types of PSGs. Table S7. The association between participation (patient or family) and all scores of MMHLQ divided by PSGs. Table S8. The association between participation (patient or family) and all scores of MMHLQ divided by types of PSGs. Table S9. The association between age (<65 y/o or ≥65 y/o) and all scores of MMHLQ divided by PSGs. Table S10. The association between age (<65 y/o or ≥65 y/o) and all scores of MMHLQ divided by types of PSGs.
