# Supplementary material for: Health Literacy Varies According to Different Background Disease Natures and Characteristics of Participants for Patient Support Groups
Source: Int J Environ Res Public Health. 2020 Aug 7;17(16):5702. doi: 10.3390/ijerph17165702 (PMC7460350; doi:10.3390/ijerph17165702)
Supplement: Supplementary file 1 [file ijerph-17-05702-s001.pdf]

Supplementary data

Table S1. Questionnaire of MMHLQ

	非常困難 1	困難 2	容易 3	非常容易 4
1.對我來說，尋找有關疾病的知識是……	☐	☐	☐	☐
2.對我來說，取得有關日常生活保健的資訊是……	☐	☐	☐	☐
3.對我來說，從網路找尋需要的健康資訊是……	☐	☐	☐	☐
4.對我來說，拿到健康檢查報告後，進一步收集相關資訊是……	☐	☐	☐	☐
5.對我來說，瞭解藥袋上的說明是……	☐	☐	☐	☐
6.對我來說，能按照醫療人員指示照護疾病是……	☐	☐	☐	☐
7.對我來說，瞭解醫療人員的說明是……	☐	☐	☐	☐
8.對我來說，能按照藥袋說明使用藥物是……	☐	☐	☐	☐
9.對我來說，判斷取得的健康資訊能不能解決健康問題是……	☐	☐	☐	☐
10.對我來說，判斷取得的健康資訊適不適合自己是……	☐	☐	☐	☐
11.對我來說，判斷取得的健康資訊跟其他資訊有沒有一致是……	☐	☐	☐	☐
12.對我來說，判斷網路的健康資訊可不可信是……	☐	☐	☐	☐
13.對我來說，應用健康資訊來瞭解病情的變化是……	☐	☐	☐	☐
14.對我來說，應用健康資訊來做好面對疾病的準備是……	☐	☐	☐	☐
15.對我來說，應用健康資訊來瞭解健康檢查結果是……	☐	☐	☐	☐
16.對我來說，應用健康資訊來選擇治療方法是……	☐	☐	☐	☐
17.對我來說，向醫師提出自己想要的檢查或治療方法是……	☐	☐	☐	☐
18.對我來說，跟醫療人員確認自己對醫療指示的理解是否正確是…	☐	☐	☐	☐
19.對我來說，跟醫師討論治療方法是……	☐	☐	☐	☐
20.對我來說，對醫療人員的說明有疑問時，能提出問題是……	☐	☐	☐	☐

Questionnaire of MMHLQ (English version)

	Very difficult	Difficult	Easy	Very easy
	1	2	3	4
1.Searching information about disease				
2.Get information about health protection				
3.Find health information from network				
4. Get information about report of health examination report				
5. Understand the instruction of medication bag				
6. Obey the instruction of medical personnel to care disease				
7. Understand the introduction of medical personnel				
8. Follow the instruction of medical bag to take medication				
9. Evaluate whether the health information can be used to solve medical problems				
10. Evaluate the health information suitable for himself/herself or not				
11. Evaluate the difference or consistence of health information				
12. Evaluate the reliability of medical information from network				
13. Apply health information to know the progress of disease				
14. Apply health information to prevent disease				
15. Apply health information to understand the report of health examination				
16. Apply health information to decided how treat disease				
17. Talk to doctors the chosen examination and treatment				
18. Make sure with medical personnel about accuracy of orders				
19. Discuss with doctor about the choice of treatment				
20. Ask medical personnel if you are not sure				

Table S2. Certification for the approval by authors (Mi-Hsiu Wei Ph. D et al) for analysis on 05/25/2018.

基於 **病友會與醫病共享決策對於病人與家屬健康識能之影響**

之需要（研究/方案摘要如附表），徵求授權同意使用「中文多面向健康識能量表(Mandarin Multidimensional Health Literacy Questionnaire, MMHLQ)」。

申請人瞭解並同意以下事項：

一、本同意書僅限於同意該量表使用於上述用途。

二、使用此量表時，對於題目、量尺、計分皆依量表原著的使用說明。

三、依學術論著規範，於適當處註明研究工具與參考文獻出處如下：

魏米秀、王英偉、張美娟、謝至鎠(2017)。中文多面向健康識能量表(MMHLQ)之發展。台灣公共衛生雜誌，36(6)，556-570。doi:10.6288/TJPH201736106061

申請人：**劉佳樺**

(所填寫之申請人姓名視同具效力之電子簽名)

申請日期(月/日/年)：05/16/2018

量表授權人：**魏米秀**

(代表本量表之研發團隊授權同意)

授權日期(月/日/年)：05/25/2018

Certification for the approval by authors (Mi-Hsiu Wei Ph. D et al) for analysis on 05/25/2018. (English version)

This certification was for the approval of Mandarin Multidimensional Health Literacy Questionnaire (MMHLQ) for the study of health literacy in patient support group.

This MMHLQ can only be used in the following situations

1. Only for the analysis in this study.
2. When using this MMHLQ, authors should use the introduction of this questionnaire.
3. When using this MMHLQ, authors should cite the original reference (as below)

Mi-Hsiu Wei Y-WW, Mei-Chuan Chang, Jyh -Gang Hsieh: Development of Mandarin Multidimensional Health Literacy Questionnaire (MMHLQ). Taiwan Journal of Public Health 2017, 36(6):556-570. Doi:10.6288/TJPH201736106061

Applicant: Chia-Hua Liou
Date of application:May/16/2018

Consenter: Mi-Hsiu Wei
Date of approval: May/25/2018

Table S3. Detailed score of MMHLQ divided by PSGs. (N=458)

	SLE	Head-and-neck cancer	DM	ADPKD	Hemodialysis	CKD	COPD	Osteoporosis	Total	*p* value
**Subscale 1: Accessing**										
1. Searching information about disease										**0.008**
Very difficult	1	2	5	1	0	3	1	1	14	
	(1.8%)	(4.7%)	(7.1%)	(1.2%)	(0.0%)	(7.9%)	(2.2%)	(1.2%)	(3.1%)	
Difficult	13	16	10	19	10	5	16	28	117	
	(23.2%)	(37.2%)	(14.3%)	(23.5%)	(23.3%)	(13.2%)	(35.6%)	(34.1%)	(25.5%)	
Easy	36	24	46	51	31	20	24	46	278	
	(64.3%)	(55.8%)	(65.7%)	(63.0%)	(72.1%)	(52.6%)	(53.3%)	(56.1%)	(60.7%)	
Very easy	6	1	9	10	2	10	4	7	49	
	(10.7%)	(2.3%)	(12.9%)	(12.3%)	(4.7%)	(26.3%)	(8.9%)	(8.5%)	(10.7%)	
2. Get information about health protection										0.061
Very difficult	0	0	3	1	0	2	2	1	9	
	(0.0%)	(0.0%)	(4.3%)	(1.2%)	(0.0%)	(5.3%)	(4.4%)	(1.2%)	(2.0%)	
Difficult	12	10	10	10	8	5	5	20	80	
	(21.4%)	(23.3%)	(14.3%)	(12.3%)	(18.6%)	(13.2%)	(11.1%)	(24.4%)	(17.5%)	
Easy	38	33	48	62	32	21	32	52	318	
	(67.9%)	(76.7%)	(68.6%)	(76.5%)	(74.4%)	(55.3%)	(71.1%)	(63.4%)	(69.4%)	
Very easy	6	0	9	8	3	10	6	9	51	
	(10.7%)	(0.0%)	(12.9%)	(9.9%)	(7.0%)	(26.3%)	(13.3%)	(11.0%)	(11.1%)	

Table S3. Detailed score of MMHLQ divided by PSGs. (N=458) (Cont.)

	SLE	Head-and-neck cancer	DM	ADPKD	Hemodialysis	CKD	COPD	Osteoporosis	Total	*p* value
3. Find health information from network										0.133
Very difficult	0 (0.0%)	2 (4.7%)	5 (7.1%)	3 (3.7%)	3 (7.0%)	3 (7.9%)	3 (6.7%)	5 (6.1%)	24 (5.2%)	
Difficult	9 (16.1%)	7 (16.3%)	18 (25.7%)	10 (12.3%)	5 (11.6%)	4 (10.5%)	7 (15.6%)	16 (19.5%)	76 (16.6%)	
Easy	35 (62.5%)	32 (74.4%)	36 (51.4%)	57 (70.4%)	31 (72.1%)	20 (52.6%)	31 (68.9%)	51 (62.2%)	293 (64.0%)	
Very easy	12 (21.4%)	2 (4.7%)	11 (15.7%)	11 (13.6%)	4 (9.3%)	11 (28.9%)	4 (8.9%)	10 (12.2%)	65 (14.2%)	
4 Get information about report of health examination report										**0.001**
Very difficult	0 (0.0%)	2 (4.7%)	2 (2.9%)	2 (2.5%)	0 (0.0%)	2 (5.3%)	1 (2.2%)	1 (1.2%)	10 (2.2%)	
Difficult	18 (32.1%)	10 (23.3%)	13 (18.6%)	12 (14.8%)	14 (32.6%)	4 (10.5%)	11 (24.4%)	27 (32.9%)	109 (23.8%)	
Easy	36 (64.3%)	31 (72.1%)	47 (67.1%)	58 (71.6%)	27 (62.8%)	20 (52.6%)	28 (62.2%)	48 (58.5%)	295 (64.4%)	
Very easy	2 (3.6%)	0 (0.0%)	8 (11.4%)	9 (11.1%)	2 (4.7%)	12 (31.6%)	5 (11.1%)	6 (7.3%)	44 (9.6%)	

Table S3. Detailed score of MMHLQ divided by PSGs. (N=458) (Cont.)

	SLE	Head-and-neck cancer	DM	ADPKD	Hemodialysis	CKD	COPD	Osteoporosis	Total	*p* value
**Subscale 2:Understanding**										
5. Understand the instruction of medication bag										0.527
Very difficult	2 (3.6%)	0 (0.0%)	1 (1.4%)	1 (1.2%)	0 (0.0%)	1 (2.6%)	0 (0.0%)	1 (1.2%)	6 (1.3%)	
Difficult	4 (7.1%)	5 (11.6%)	6 (8.6%)	11 (13.6%)	3 (7.0%)	3 (7.9%)	6 (13.3%)	11 (13.4%)	49 (10.7%)	
Easy	42 (75.0%)	35 (81.4%)	54 (77.1%)	54 (66.7%)	36 (83.7%)	24 (63.2%)	29 (64.4%)	61 (74.4%)	335 (73.1%)	
Very easy	8 (14.3%)	3 (7.0%)	9 (12.9%)	15 (18.5%)	4 (9.3%)	10 (26.3%)	10 (22.2%)	9 (11.0%)	68 (14.8%)	
6. Obey the instruction of medical personnel to care disease										0.359
Very difficult	2 (3.6%)	0 (0.0%)	1 (1.4%)	1 (1.2%)	0 (0.0%)	1 (2.6%)	0 (0.0%)	1 (1.2%)	6 (1.3%)	
Difficult	4 (7.1%)	9 (20.9%)	8 (11.4%)	9 (11.1%)	6 (14.0%)	4 (10.5%)	2 (4.4%)	5 (6.1%)	47 (10.3%)	
Easy	37 (66.1%)	31 (72.1%)	49 (70.0%)	57 (70.4%)	31 (72.1%)	22 (57.9%)	35 (77.8%)	64 (78.0%)	326 (71.2%)	
Very easy	13 (23.2%)	3 (7.0%)	12 (17.1%)	14 (17.3%)	6 (14.0%)	11 (28.9%)	8 (17.8%)	12 (14.6%)	79 (17.2%)	

Table S3. Detailed score of MMHLQ divided by PSGs. (N=458) (Cont.)

	SLE	Head-and-neck cancer	DM	ADPKD	Hemodialysis	CKD	COPD	Osteoporosis	Total	*p* value
7. Understand the introduction of medical personnel										0.318
Very difficult	0<br>(0.0%)	0<br>(0.0%)	2<br>(2.9%)	0<br>(0.0%)	0<br>(0.0%)	1<br>(2.6%)	0<br>(0.0%)	1<br>(1.2%)	4<br>(0.9%)	
Difficult	3<br>(5.4%)	6<br>(14.0%)	4<br>(5.7%)	9<br>(11.1%)	3<br>(7.0%)	3<br>(7.9%)	1<br>(2.2%)	4<br>(4.9%)	33<br>(7.2%)	
Easy	43<br>(76.8%)	35<br>(81.4%)	54<br>(77.1%)	59<br>(72.8%)	35<br>(81.4%)	24<br>(63.2%)	34<br>(75.6%)	65<br>(79.3%)	349<br>(76.2%)	
Very easy	10<br>(17.9%)	2<br>(4.7%)	10<br>(14.3%)	13<br>(16.0%)	5<br>(11.6%)	10<br>(26.3%)	10<br>(22.2%)	12<br>(14.6%)	72<br>(15.7%)	
8. Follow the instruction of medical bag to take medication										0.685
Very difficult	1<br>(1.8%)	0<br>(0.0%)	1<br>(1.4%)	0<br>(0.0%)	0<br>(0.0%)	0<br>(0.0%)	0<br>(0.0%)	1<br>(1.2%)	3<br>(0.7%)	
Difficult	4<br>(7.3%)	1<br>(2.3%)	7<br>(10.0%)	10<br>(12.3%)	1<br>(2.3%)	2<br>(5.3%)	2<br>(4.4%)	7<br>(8.5%)	34<br>(7.4%)	
Easy	39<br>(70.9%)	38<br>(88.4%)	49<br>(70.0%)	54<br>(66.7%)	34<br>(79.1%)	26<br>(68.4%)	32<br>(71.1%)	56<br>(68.3%)	328<br>(71.8%)	
Very easy	11<br>(20.0%)	4<br>(9.3%)	13<br>(18.6%)	17<br>(21.0%)	8<br>(18.6%)	10<br>(26.3%)	11<br>(24.4%)	18<br>(22.0%)	92<br>(20.1%)	

Table S3. Detailed score of MMHLQ divided by PSGs. (N=458) (Cont.)

	SLE	Head-and-neck cancer	DM	ADPKD	Hemodialysis	CKD	COPD	Osteoporosis	Total	*p* value
**Subscale 3:Appraisal**										
9. Evaluate whether the health information can be used to solve medical problems										**0.004**
Very difficult	1 (1.8%)	0 (0.0%)	1 (1.4%)	3 (3.7%)	1 (2.3%)	0 (0.0%)	0 (0.0%)	4 (4.9%)	10 (2.2%)	
Difficult	16 (28.6%)	18 (41.9%)	19 (27.1%)	24 (29.6%)	13 (30.2%)	12 (31.6%)	10 (22.2%)	32 (39.0%)	144 (31.4%)	
Easy	37 (66.1%)	25 (58.1%)	44 (62.9%)	47 (58.0%)	27 (62.8%)	16 (42.1%)	30 (66.7%)	44 (53.7%)	270 (59.0%)	
Very easy	2 (3.6%)	0 (0.0%)	6 (8.6%)	7 (8.6%)	2 (4.7%)	10 (26.3%)	5 (11.1%)	2 (2.4%)	34 (7.4%)	
10. Evaluate the health information suitable for himself/herself or not										**0.016**
Very difficult	2 (3.6%)	0 (0.0%)	2 (2.9%)	2 (2.5%)	1 (2.3%)	0 (0.0%)	0 (0.0%)	3 (3.7%)	10 (2.2%)	
Difficult	17 (30.4%)	18 (41.9%)	20 (28.6%)	20 (24.7%)	13 (30.2%)	9 (23.7%)	10 (22.2%)	31 (37.8%)	138 (30.1%)	
Easy	35 (62.5%)	24 (55.8%)	42 (60.0%)	53 (65.4%)	25 (58.1%)	19 (50.0%)	30 (66.7%)	47 (57.3%)	275 (60.0%)	
Very easy	2 (3.6%)	1 (2.3%)	6 (8.6%)	6 (7.4%)	4 (9.3%)	10 (26.3%)	5 (11.1%)	1 (1.2%)	35 (7.6%)	

Table S3. Detailed score of MMHLQ divided by PSGs. (N=458) (Cont.)

	SLE	Head-and-neck cancer	DM	ADPKD	Hemodialysis	CKD	COPD	Osteoporosis	Total	*p* value
11. Evaluate the difference or consistence of health information										**<0.001**
Very difficult	4 (7.1%)	0 (0.0%)	2 (2.9%)	2 (2.5%)	3 (7.0%)	1 (2.6%)	1 (2.2%)	4 (4.9%)	17 (3.7%)	
Difficult	23 (41.1%)	16 (37.2%)	24 (34.3%)	25 (30.9%)	10 (23.3%)	12 (31.6%)	9 (20.0%)	37 (45.1%)	156 (34.1%)	
Easy	29 (51.8%)	26 (60.5%)	37 (52.9%)	45 (55.6%)	30 (69.8%)	15 (39.5%)	30 (66.7%)	40 (48.8%)	252 (55.0%)	
Very easy	0 (0.0%)	1 (2.3%)	7 (10.0%)	9 (11.1%)	0 (0.0%)	10 (26.3%)	5 (11.1%)	1 (1.2%)	33 (7.2%)	
12. Evaluate the reliability of medical										**0.001**
Very difficult	4 (7.1%)	2 (4.7%)	6 (8.6%)	2 (2.5%)	3 (7.0%)	2 (5.3%)	2 (4.4%)	7 (8.5%)	28 (6.1%)	
Difficult	22 (39.3%)	14 (32.6%)	27 (38.6%)	29 (35.8%)	15 (34.9%)	12 (31.6%)	14 (31.1%)	46 (56.1%)	179 (39.1%)	
Easy	29 (51.8%)	26 (60.5%)	33 (47.1%)	42 (51.9%)	22 (51.2%)	14 (36.8%)	26 (57.8%)	27 (32.9%)	219 (47.8%)	
Very easy	1 (1.8%)	1 (2.3%)	4 (5.7%)	8 (9.9%)	3 (7.0%)	10 (26.3%)	3 (6.7%)	2 (2.4%)	32 (7.0%)	

Table S3. Detailed score of MMHLQ divided by PSGs. (N=458) (Cont.)

	SLE	Head-and-neck cancer	DM	ADPKD	Hemodialysis	CKD	COPD	Osteoporosis	Total	*p* value
**Subscale 4:Application**										
13. Apply health information to know the progress of disease										**<0.001**
Very difficult	1 (1.8%)	3 (7.0%)	1 (1.4%)	1 (1.2%)	0 (0.0%)	0 (0.0%)	0 (0.0%)	1 (1.2%)	7 (1.5%)	
Difficult	19 (33.9%)	10 (23.3%)	15 (21.4%)	24 (29.6%)	13 (30.2%)	9 (23.7%)	14 (31.1%)	34 (41.5%)	138 (30.1%)	
Easy	33 (58.9%)	30 (69.8%)	50 (71.4%)	48 (59.3%)	28 (65.1%)	17 (44.7%)	26 (57.8%)	47 (57.3%)	279 (60.9%)	
Very easy	3 (5.4%)	0 (0.0%)	4 (5.7%)	8 (9.9%)	2 (4.7%)	12 (31.6%)	5 (11.1%)	0 (0.0%)	34 (7.4%)	
14. Apply health information to prevent disease										**<0.001**
Very difficult	0 (0.0%)	1 (2.3%)	2 (2.9%)	1 (1.2%)	0 (0.0%)	0 (0.0%)	1 (2.2%)	1 (1.2%)	6 (1.3%)	
Difficult	14 (25.0%)	14 (32.6%)	11 (15.7%)	21 (25.9%)	7 (16.3%)	7 (18.4%)	6 (13.3%)	29 (35.4%)	109 (23.8%)	
Easy	38 (67.9%)	28 (65.1%)	54 (77.1%)	50 (61.7%)	36 (83.7%)	21 (55.3%)	33 (73.3%)	49 (59.8%)	309 (67.5%)	
Very easy	4 (7.1%)	0 (0.0%)	3 (4.3%)	9 (11.1%)	0 (0.0%)	10 (26.3%)	5 (11.1%)	3 (3.7%)	34 (7.4%)	

Table S3. Detailed score of MMHLQ divided by PSGs. (N=458) (Cont.)

	SLE	Head-and-neck cancer	DM	ADPKD	Hemodialysis	CKD	COPD	Osteoporosis	Total	*p* value
15. Apply health information to understand the report of health examination										**0.002**
Very difficult	0<br>(0.0%)	2<br>(4.7%)	1<br>(1.4%)	1<br>(1.2%)	0<br>(0.0%)	0<br>(0.0%)	0<br>(0.0%)	2<br>(2.4%)	6<br>(1.3%)	
Difficult	14<br>(25.0%)	9<br>(20.9%)	8<br>(11.4%)	16<br>(19.8%)	5<br>(11.6%)	4<br>(10.5%)	13<br>(28.9%)	22<br>(26.8%)	91<br>(19.9%)	
Easy	37<br>(66.1%)	32<br>(74.4%)	58<br>(82.9%)	55<br>(67.9%)	36<br>(83.7%)	24<br>(63.2%)	28<br>(62.2%)	54<br>(65.9%)	324<br>(70.7%)	
Very easy	5<br>(8.9%)	0<br>(0.0%)	3<br>(4.3%)	9<br>(11.1%)	2<br>(4.7%)	10<br>(26.3%)	4<br>(8.9%)	4<br>(4.9%)	37<br>(8.1%)	
16. Apply health information to decided how treat disease										**0.001**
Very difficult	1<br>(1.8%)	1<br>(2.3%)	1<br>(1.4%)	1<br>(1.2%)	0<br>(0.0%)	0<br>(0.0%)	0<br>(0.0%)	2<br>(2.4%)	6<br>(1.3%)	
Difficult	16<br>(28.6%)	19<br>(44.2%)	13<br>(18.6%)	24<br>(29.6%)	10<br>(23.3%)	7<br>(18.4%)	13<br>(28.9%)	29<br>(35.4%)	131<br>(28.6%)	
Easy	35<br>(62.5%)	23<br>(53.5%)	52<br>(74.3%)	49<br>(60.5%)	32<br>(74.4%)	21<br>(55.3%)	27<br>(60.0%)	51<br>(62.2%)	290<br>(63.3%)	
Very easy	4<br>(7.1%)	0<br>(0.0%)	4<br>(5.7%)	7<br>(8.6%)	1<br>(2.3%)	10<br>(26.3%)	5<br>(11.1%)	0<br>(0.0%)	31<br>(6.8%)	

Table S3. Detailed score of MMHLQ divided by PSGs. (N=458) (Cont.)

	SLE	Head-and-neck cancer	DM	ADPKD	Hemodialysis	CKD	COPD	Osteoporosis	Total	*p* value
**Subscale 5:Communication**										
17. Talk to doctors the chosen examination and treatment										**<0.001**
Very difficult	2 (3.6%)	0 (0.0%)	2 (2.9%)	0 (0.0%)	0 (0.0%)	0 (0.0%)	1 (2.2%)	4 (4.9%)	9 (2.0%)	
Difficult	11 (19.6%)	13 (30.2%)	14 (20.0%)	15 (18.5%)	9 (20.9%)	4 (10.5%)	6 (13.3%)	29 (35.4%)	101 (22.1%)	
Easy	36 (64.3%)	28 (65.1%)	50 (71.4%)	56 (69.1%)	33 (76.7%)	22 (57.9%)	34 (75.6%)	48 (58.5%)	307 (67.0%)	
Very easy	7 (12.5%)	2 (4.7%)	4 (5.7%)	10 (12.3%)	1 (2.3%)	12 (31.6%)	4 (8.9%)	1 (1.2%)	41 (9.0%)	
18. Make sure with medical personnel about accuracy of orders										**0.001**
Very difficult	0 (0.0%)	3 (7.0%)	1 (1.4%)	0 (0.0%)	0 (0.0%)	0 (0.0%)	0 (0.0%)	3 (3.7%)	7 (1.5%)	
Difficult	7 (12.5%)	7 (16.3%)	11 (15.7%)	18 (22.2%)	6 (14.0%)	2 (5.3%)	7 (15.6%)	19 (23.2%)	77 (16.8%)	
Easy	44 (78.6%)	30 (69.8%)	55 (78.6%)	53 (65.4%)	34 (79.1%)	25 (65.8%)	34 (75.6%)	57 (69.5%)	332 (72.5%)	
Very easy	5 (8.9%)	3 (7.0%)	3 (4.3%)	10 (12.3%)	3 (7.0%)	11 (28.9%)	4 (8.9%)	3 (3.7%)	42 (9.2%)	

Table S3. Detailed score of MMHLQ divided by PSGs. (N=458) (Cont.)

	SLE	Head-and-neck cancer	DM	ADPKD	Hemodialysis	CKD	COPD	Osteoporosis	Total	*p* value
19. Make sure with medical personnel about accuracy of orders										**0.008**
Very difficult	0<br>(0.0%)	0<br>(0.0%)	1<br>(1.4%)	1<br>(1.2%)	0<br>(0.0%)	0<br>(0.0%)	0<br>(0.0%)	3<br>(3.7%)	5<br>(1.1%)	
Difficult	6<br>(10.7%)	11<br>(25.6%)	11<br>(15.7%)	15<br>(18.5%)	8<br>(18.6%)	7<br>(18.4%)	3<br>(6.7%)	19<br>(23.2%)	80<br>(17.5%)	
Easy	41<br>(73.2%)	27<br>(62.8%)	54<br>(77.1%)	54<br>(66.7%)	32<br>(74.4%)	20<br>(52.6%)	34<br>(75.6%)	58<br>(70.7%)	320<br>(69.9%)	
Very easy	9<br>(16.1%)	5<br>(11.6%)	4<br>(5.7%)	11<br>(13.6%)	3<br>(7.0%)	11<br>(28.9%)	8<br>(17.8%)	2<br>(2.4%)	53<br>(11.6%)	
20. Ask medical personnel if you are not sure										**0.002**
Very difficult	0<br>(0.0%)	1<br>(2.3%)	2<br>(2.9%)	1<br>(1.2%)	0<br>(0.0%)	0<br>(0.0%)	0<br>(0.0%)	1<br>(1.2%)	5<br>(1.1%)	
Difficult	4<br>(7.1%)	8<br>(18.6%)	9<br>(12.9%)	12<br>(14.8%)	5<br>(11.6%)	3<br>(7.9%)	6<br>(13.3%)	24<br>(29.3%)	71<br>(15.5%)	
Easy	49<br>(87.5%)	31<br>(72.1%)	55<br>(78.6%)	57<br>(70.4%)	35<br>(81.4%)	24<br>(63.2%)	31<br>(68.9%)	52<br>(63.4%)	334<br>(72.9%)	
Very easy	3<br>(5.4%)	3<br>(7.0%)	4<br>(5.7%)	11<br>(13.6%)	3<br>(7.0%)	11<br>(28.9%)	8<br>(17.8%)	5<br>(6.1%)	48<br>(10.5%)	

Table S4. Background characteristics of participants and score of MMHLQ divided by **types** of PSGs (N=458)

	Autoimmune disease	Malignancy	Chronic disease	Genetic disease	Degenerative disease	Total	*p* value
Case (n, %)	56 (93.3%)	43 (86.0%)	196 (87.1%)	81 (90.0%)	82 (91.1%)	458 (91.1%)	
1st time participation (n, %)	31 (55.4%)	31 (72.1%)	125 (63.8%)	65 (80.2%)	80 (97.6%)	332 (72.5%)	**<0.001**
Patient (n, %), not family	34 (60.7%)	27 (62.8%)	133 (67.9%)	27 (33.3%)	59 (72.0%)	280 (61.1%)	**<0.001**
Age (y/o)	42.21 ±16.49	53.40 ±13.37	59.03 ±15.28	48.95 ±17.58	62.60 ±11.05	55.30 ±16.39	**<0.001**
MMHLQ							
**Subscale 1: Accessing**							
1.Searching information about disease	2.84 ±0.63	2.56 ±0.63	2.83 ±0.70	2.86 ±0.63	2.72 ±0.63	2.79 ±0.66	0.089
2.Get information about health protection	2.89 ±0.56	2.77 ±0.43	2.93 ±0.65	2.95 ±0.52	2.84 ±0.62	2.90 ±0.60	0.413
3.Find health information from network	3.05 ±0.62	2.79 ±0.60	2.84 ±0.77	2.94 ±0.64	2.80 ±0.73	2.87 ±0.71	0.186
4. Get information about report of health examination report	2.71 ±0.53	2.67 ±0.57	2.87 ±0.66	2.91 ±0.60	2.72 ±0.61	2.81 ±0.62	0.054
Total	11.50 ±1.82	10.79 ±1.86	11.46 ±2.45	11.67 ±2.17	11.09 ±2.14	11.37 ±2.23	0.185

Table S4. Background characteristics of participants and score of MMHLQ divided by **types** of PSGs (N=458) (Cont.)

	Autoimmune disease	Malignancy	Chronic disease	Genetic disease	Degenerative disease	Total	*p* value
**Subscale 2: Understanding**							
5. Understand the instruction of medication bag	3.00 ±0.60	2.95 ±0.43	3.06 ±0.55	3.02 ±0.61	2.95 ±0.54	3.02 ±0.56	0.602
6. Obey the instruction of medical personnel to care disease	3.09 ±0.67	2.86 ±0.52	3.07 ±0.57	3.04 ±0.58	3.06 ±0.51	3.04 ±0.57	0.270
7. Understand the introduction of medical personnel	3.13 ±0.47	2.91 ±0.43	3.09 ±0.54	3.05 ±0.52	3.07 ±0.49	3.07 ±0.51	0.237
8. Follow the instruction of medical bag to take medication	3.14 ±0.70	3.07 ±0.34	3.14 ±0.53	3.09 ±0.57	3.11 ±0.59	3.12 ±0.55	0.884
Total	12.36 ±1.92	11.79 ±1.21	12.36 ±1.92	12.20 ±2.08	12.20 ±1.69	12.25 ±1.85	0.462
**Subscale 3: Appraisal**							
9. Evaluate whether the health information can be used to solve medical problems	2.71 ±0.56	2.58 ±0.50	2.82 ±0.64	2.72 ±0.68	2.54 ±0.63	2.72 ±0.63	**0.007**
10. Evaluate the health information suitable for himself/herself or not	2.66 ±0.61	2.60 ±0.54	2.83 ±0.65	2.78 ±0.61	2.56 ±0.59	2.73 ±0.63	**0.007**
11. Evaluate the difference or consistence of health information	2.45 ±0.63	2.65 ±0.53	2.76 ±0.69	2.75 ±0.68	2.46 ±0.61	2.66 ±0.67	**0.001**
12. Evaluate the reliability of medical information from network	2.48 ±0.66	2.60 ±0.62	2.62 ±0.76	2.69 ±0.68	2.29 ±0.66	2.56 ±0.71	**0.002**
Total	10.30 ±2.04	10.44 ±1.84	11.04 ±2.49	10.94 ±2.40	9.85 ±2.07	10.66 ±2.33	**0.001**

Table S4. Background characteristics of participants and score of MMHLQ divided by **types** of PSGs (N=458) (Cont.)

	Autoimmune disease	Malignancy	Chronic disease	Genetic disease	Degenerative disease	Total	*p* value
**Subscale 4: Application**							
13. Apply health information to know the progress of disease	2.68 ±0.61	2.63 ±0.62	2.85 ±0.61	2.78 ±0.63	2.56 ±0.52	2.74 ±0.61	**0.004**
14. Apply health information to prevent disease	2.82 ±0.54	2.63 ±0.54	2.90 ±0.55	2.83 ±0.63	2.66 ±0.57	2.81 ±0.57	**0.004**
15. Apply health information to understand the report of health examination	2.84 ±0.56	2.70 ±0.56	2.93 ±0.52	2.89 ±0.59	2.73 ±0.59	2.86 ±0.56	**0.020**
16. Apply health information to decided how treat disease	2.75 ±0.61	2.51 ±0.55	2.87 ±0.57	2.77 ±0.62	2.60 ±0.54	2.76 ±0.59	**<0.001**
Total	11.09 ±1.84	10.47 ±1.83	11.56 ±2.00	11.26 ±2.33	10.55 ±1.87	11.16 ±2.04	**<0.001**
**Subscale 5: Communication**							
17. Talk to doctors the chosen examination and treatment	2.86 ±0.67	2.74 ±0.54	2.91 ±0.57	2.94 ±0.56	2.56 ±0.61	2.83 ±0.60	**<0.001**
18. Make sure with medical personnel about accuracy of orders	2.96 ±0.47	2.77 ±0.68	2.96 ±0.51	2.90 ±0.58	2.73 ±0.59	2.89 ±0.56	**0.010**
19. Discuss with doctor about the choice of treatment	3.05 ±0.52	2.86 ±0.60	2.97 ±0.55	2.93 ±0.61	2.72 ±0.57	2.92 ±0.57	**0.004**
20. Ask medical personnel if you are not sure	2.98 ±0.36	2.84 ±0.57	2.99 ±0.54	2.96 ±0.58	2.74 ±0.58	2.93 ±0.55	**0.006**
Total	11.86 ±1.69	11.21 ±2.05	11.84 ±1.92	11.73 ±2.15	10.76 ±1.96	11.57 ±1.99	**<0.001**

Table S5 The association between first time participation and **all scores** of MMHLQ **divided by PSGs**

	SLE			Head-and-neck cancer			DM			ADPKD			Hemodialysis			CKD			COPD			Osteoporosis		
	1st time	Not 1st time	*p* value	1st time	Not 1st time	*p* value	1st time	Not 1st time	*p* value	1st time	Not 1st time	*p* value	1st time	Not 1st time	*p* value	1st time	Not 1st time	*p* value	1st time	Not 1st time	*p* value	1st time	Not 1st time	*p* value
**Accessing**																								
1.Searching information about disease	2.81 ±0.60	2.88 ±0.67	0.666	2.65 ±0.55	2.33 ±0.78	0.147	2.81 ±0.79	2.89 ±0.64	0.681	2.85 ±0.67	2.94 ±0.44	0.513	2.67 ±0.48	2.95 ±0.49	0.058	3.03 ±0.85	2.60 ±0.89	0.300	2.68 ±0.72	2.71 ±0.59	0.896	2.71 ±0.62	3.00 ±1.41	0.822
2.Get information about health protection	2.84 ±0.52	2.96 ±0.61	0.427	2.84 ±0.37	2.58 ±0.51	0.137	2.91 ±0.68	2.89 ±0.64	0.912	2.94 ±0.56	3.00 ±0.37	0.675	2.71 ±0.56	3.05 ±0.38	**0.030**	3.06 ±0.83	2.80 ±0.45	0.498	2.89 ±0.74	3.00 ±0.50	0.600	2.83 ±0.61	3.50 ±0.71	0.128
3.Find health information from network	3.13 ±0.67	2.96 ±0.54	0.311	2.94 ±0.44	2.42 ±0.79	0.051	2.81 ±0.85	2.67 ±0.73	0.461	2.91 ±0.68	3.06 ±0.44	0.389	2.90 ±0.44	2.77 ±0.87	0.531	3.03 ±0.88	3.00 ±0.71	0.942	3.00 ±0.61	2.47 ±0.72	**0.017**	2.79 ±0.72	3.50 ±0.71	0.173
4. Get information about report of health examination report	2.74 ±0.58	2.68 ±0.48	0.668	2.77 ±0.43	2.42 ±0.79	0.161	2.88 ±0.70	2.85 ±0.53	0.840	2.89 ±0.62	3.00 ±0.52	0.521	2.62 ±0.59	2.82 ±0.50	0.239	3.15 ±0.83	2.80 ±0.45	0.366	2.82 ±0.72	2.82 ±0.53	0.992	2.70 ±0.60	3.50 ±0.71	0.069
Total	11.52 ±1.77	11.48 ±1.92	0.942	11.19 ±1.35	9.75 ±2.56	0.086	11.42 ±2.77	11.30 ±2.32	0.849	11.58 ±2.30	12.00 ±1.59	0.497	10.90 ±1.70	11.59 ±1.53	0.172	12.27 ±3.29	11.20 ±1.92	0.485	11.39 ±2.51	11.00 ±1.58	0.567	11.03 ±2.09	13.50 ±3.54	0.106

Table S5 The association between first time participation and **all scores** of MMHLQ **divided by PSGs** (Cont.)

	SLE			Head-and-neck cancer			DM			ADPKD			Hemodialysis			CKD			COPD			Osteoporosis		
	1st time	Not 1st time	*p* value	1st time	Not 1st time	*p* value	1st time	Not 1st time	*p* value	1st time	Not 1st time	*p* value	1st time	Not 1st time	*p* value	1st time	Not 1st time	*p* value	1st time	Not 1st time	*p* value	1st time	Not 1st time	*p* value
**Understanding**																								
5. Understand the instruction of medication bag	2.97 ±0.66	3.04 ±0.54	0.660	3.00 ±0.37	2.83 ±0.58	0.367	3.00 ±0.58	3.04 ±0.44	0.776	3.03 ±0.64	3.00 ±0.52	0.858	2.95 ±0.38	3.09 ±0.43	0.270	3.18 ±0.68	2.80 ±0.45	0.236	3.11 ±0.63	3.06 ±0.56	0.796	2.94 ±0.54	3.50 ±0.71	0.148
6. Obey the instruction of medical personnel to care disease	3.10 ±0.65	3.08 ±0.70	0.927	2.94 ±0.51	2.67 ±0.49	0.127	3.02 ±0.64	3.04 ±0.52	0.925	3.05 ±0.57	3.00 ±0.63	0.777	2.90 ±0.54	3.09 ±0.53	0.259	3.21 ±0.70	2.60 ±0.55	0.069	3.14 ±0.52	3.12 ±0.33	0.860	3.05 ±0.50	3.50 ±0.71	0.216
7. Understand the introduction of medical personnel	3.10 ±0.47	3.16 ±0.47	0.621	2.94 ±0.44	2.83 ±0.39	0.487	3.02 ±0.64	3.04 ±0.44	0.922	3.05 ±0.51	3.06 ±0.57	0.912	2.95 ±0.50	3.14 ±0.35	0.167	3.15 ±0.71	3.00 ±0.00	0.231	3.25 ±0.52	3.12 ±0.33	0.302	3.06 ±0.49	3.50 ±0.71	0.216
8. Follow the instruction of medical bag to take medication	3.00 ±0.64	3.20 ±0.50	0.211	3.13 ±0.34	2.92 ±0.29	0.064	3.02 ±0.67	3.11 ±0.42	0.546	3.06 ±0.56	3.19 ±0.66	0.435	3.05 ±0.38	3.27 ±0.46	0.087	3.24 ±0.56	3.00 ±0.00	**0.018**	3.21 ±0.50	3.18 ±0.53	0.811	3.13 ±0.58	2.50 ±0.71	0.139
Total	12.26 ±1.95	12.48 ±1.92	0.671	12.00 ±1.06	11.25 ±1.42	0.117	12.07 ±2.19	12.22 ±1.72	0.759	12.18 ±2.10	12.25 ±2.05	0.911	11.86 ±1.39	12.59 ±1.44	0.096	12.79 ±2.53	11.40 ±0.55	**0.010**	12.71 ±1.94	12.47 ±1.28	0.648	12.18 ±1.67	13.00 ±2.83	0.498

Table S5 The association between first time participation and **all scores** of MMHLQ **divided by PSGs** (Cont.)

	SLE			Head-and-neck cancer			DM			ADPKD			Hemodialysis			CKD			COPD			Osteoporosis		
	1st time	Not 1st time	*p* value	1st time	Not 1st time	*p* value	1st time	Not 1st time	*p* value	1st time	Not 1st time	*p* value	1st time	Not 1st time	*p* value	1st time	Not 1st time	*p* value	1st time	Not 1st time	*p* value	1st time	Not 1st time	*p* value
**Evaluation**																								
9. Evaluate whether the health information can be used to solve medical problems	2.74 ±0.63	2.68 ±0.48	0.686	2.65 ±0.49	2.42 ±0.51	0.181	2.81 ±0.63	2.74 ±0.59	0.629	2.71 ±0.70	2.75 ±0.58	0.824	2.57 ±0.68	2.82 ±0.50	0.184	3.06 ±0.75	2.20 ±0.45	0.018	2.75 ±0.59	3.12 ±0.49	**0.035**	2.54 ±0.64	2.50 ±0.71	0.935
10. Evaluate the health information suitable for himself/herself or not	2.71 ±0.64	2.60 ±0.58	0.505	2.65 ±0.55	2.50 ±0.52	0.436	2.79 ±0.64	2.67 ±0.68	0.443	2.77 ±0.63	2.81 ±0.54	0.802	2.67 ±0.66	2.82 ±0.66	0.457	3.09 ±0.72	2.60 ±0.55	0.156	2.75 ±0.65	3.12 ±0.33	**0.016**	2.56 ±0.59	2.50 ±0.71	0.883
11. Evaluate the difference or consistence of health information	2.35 ±0.71	2.56 ±0.51	0.213	2.71 ±0.53	2.50 ±0.52	0.249	2.77 ±0.68	2.59 ±0.69	0.304	2.72 ±0.70	2.88 ±0.62	0.427	2.52 ±0.60	2.73 ±0.63	0.286	2.97 ±0.85	2.40 ±0.55	0.156	2.79 ±0.69	3.00 ±0.50	0.234	2.45 ±0.61	3.00 ±0.00	**<0.001**
12. Evaluate the reliability of medical information from network	2.45 ±0.72	2.52 ±0.59	0.704	2.68 ±0.54	2.42 ±0.79	0.311	2.47 ±0.77	2.56 ±0.70	0.621	2.63 ±0.67	2.94 ±0.68	0.108	2.52 ±0.75	2.64 ±0.73	0.620	2.94 ±0.90	2.20 ±0.45	0.082	2.57 ±0.74	2.82 ±0.53	0.192	2.28 ±0.66	3.00 ±0.00	**<0.001**
Total	10.26 ±2.18	10.36 ±1.89	0.854	10.68 ±1.68	9.83 ±2.17	0.242	10.84 ±2.39	10.56 ±2.44	0.635	10.83 ±2.50	11.38 ±1.96	0.420	10.29 ±2.39	11.00 ±2.12	0.305	12.06 ±3.10	9.40 ±1.67	0.071	10.86 ±2.45	12.06 ±1.52	0.048	9.83 ±2.09	11.00 ±1.41	0.432

Table S5 The association between first time participation and **all scores** of MMHLQ **divided by PSGs** (Cont.)

	SLE			Head-and-neck cancer			DM			ADPKD			Hemodialysis			CKD			COPD			Osteoporosis		
	1st time	Not 1st time	*p* value	1st time	Not 1st time	*p* value	1st time	Not 1st time	*p* value	1st time	Not 1st time	*p* value	1st time	Not 1st time	*p* value	1st time	Not 1st time	*p* value	1st time	Not 1st time	*p* value	1st time	Not 1st time	*p* value
**Application**																								
13. Apply health information to know the progress of disease	2.68 ±0.65	2.68 ±0.56	0.988	2.74 ±0.51	2.33 ±0.78	0.114	2.79 ±0.56	2.85 ±0.53	0.652	2.77 ±0.68	2.81 ±0.40	0.808	2.62 ±0.59	2.86 ±0.47	0.141	3.18 ±0.73	2.40 ±0.55	**0.028**	2.79 ±0.69	2.82 ±0.53	0.847	2.55 ±0.53	3.00 ±0.00	**<0.001**
14. Apply health information to prevent disease	2.87 ±0.50	2.76 ±0.60	0.452	2.71 ±0.53	2.42 ±0.51	0.108	2.79 ±0.60	2.89 ±0.42	0.461	2.82 ±0.63	2.88 ±0.62	0.736	2.81 ±0.40	2.86 ±0.35	0.641	3.12 ±0.70	2.80 ±0.45	0.327	2.93 ±0.60	2.94 ±0.56	0.945	2.65 ±0.58	3.00 ±0.00	**<0.001**
15. Apply health information to understand the report of health examination	2.94 ±0.51	2.72 ±0.61	0.167	2.77 ±0.43	2.50 ±0.80	0.278	2.86 ±0.52	2.96 ±0.34	0.318	2.89 ±0.62	2.88 ±0.50	0.917	2.90 ±0.30	2.95 ±0.49	0.690	3.21 ±0.60	2.80 ±0.45	0.151	2.75 ±0.65	2.88 ±0.49	0.439	2.73 ±0.59	3.00 ±0.00	**<0.001**
16. Apply health information to decided how treat disease	2.77 ±0.62	2.72 ±0.61	0.745	2.58 ±0.50	2.33 ±0.65	0.190	2.88 ±0.50	2.78 ±0.58	0.418	2.77 ±0.66	2.75 ±0.45	0.912	2.67 ±0.48	2.91 ±0.43	0.089	3.18 ±0.64	2.40 ±0.55	**0.013**	2.71 ±0.66	3.00 ±0.50	0.108	2.59 ±0.54	3.00 ±0.00	**<0.001**
Total	11.26 ±1.61	10.88 ±2.11	0.450	10.81 ±1.49	9.58 ±2.35	0.115	11.33 ±1.98	11.48 ±1.50	0.728	11.25 ±2.47	11.31 ±1.70	0.919	11.00 ±1.22	11.59 ±1.40	0.150	12.70 ±2.53	10.40 ±1.52	0.057	11.18 ±2.36	11.65 ±1.84	0.488	10.51 ±1.88	12.00 ±0.00	**<0.001**

Table S5 The association between first time participation and **all scores** of MMHLQ **divided by PSGs** (Cont.)

	SLE			Head-and-neck cancer			DM			ADPKD			Hemodialysis			CKD			COPD			Osteoporosis		
	1st time	Not 1st time	*p* value	1st time	Not 1st time	*p* value	1st time	Not 1st time	*p* value	1st time	Not 1st time	*p* value	1st time	Not 1st time	*p* value	1st time	Not 1st time	*p* value	1st time	Not 1st time	*p* value	1st time	Not 1st time	*p* value
**Communication**																								
17. Talk to doctors the chosen examination and treatment	2.84 ±0.64	2.88 ±0.73	0.822	2.81 ±0.54	2.58 ±0.51	0.227	2.79 ±0.56	2.81 ±0.62	0.867	2.94 ±0.58	2.94 ±0.44	0.995	2.71 ±0.46	2.91 ±0.43	0.159	3.33 ±0.54	2.40 ±0.55	**0.001**	2.86 ±0.65	3.00 ±0.35	0.346	2.55 ±0.61	3.00 ±0.00	**<0.001**
18. Make sure with medical personnel about accuracy of orders	3.03 ±0.48	2.88 ±0.44	0.227	2.90 ±0.60	2.42 ±0.79	0.072	2.86 ±0.52	2.85 ±0.46	0.944	2.91 ±0.61	2.88 ±0.50	0.842	2.86 ±0.36	3.00 ±0.53	0.312	3.33 ±0.48	2.60 ±0.55	**0.003**	2.89 ±0.57	3.00 ±0.35	0.439	2.73 ±0.59	3.00 ±0.00	0.518
19. Discuss with doctor about the choice of treatment	3.03 ±0.55	3.08 ±0.49	0.736	2.87 ±0.62	2.83 ±0.58	0.856	2.81 ±0.50	2.96 ±0.52	0.235	2.89 ±0.62	3.06 ±0.57	0.319	2.76 ±0.54	3.00 ±0.44	0.121	3.21 ±0.65	2.40 ±0.55	**0.012**	3.14 ±0.52	3.06 ±0.43	0.581	2.70 ±0.56	3.50 ±0.71	0.050
20. Ask medical personnel if you are not sure	2.94 ±0.36	3.04 ±0.35	0.279	2.90 ±0.54	2.67 ±0.65	0.230	2.79 ±0.56	3.00 ±0.48	0.113	2.94 ±0.58	3.06 ±0.57	0.447	2.90 ±0.30	3.00 ±0.53	0.478	3.30 ±0.53	2.60 ±0.55	**0.009**	3.04 ±0.64	3.06 ±0.43	0.895	2.73 ±0.57	3.50 ±0.71	0.063
Total	11.84 ±1.71	11.88 ±1.69	0.929	11.48 ±2.01	10.50 ±2.07	0.161	11.26 ±1.83	11.63 ±1.82	0.407	11.68 ±2.21	11.94 ±1.95	0.667	11.24 ±1.34	11.91 ±1.66	0.153	13.18 ±2.10	10.00 ±1.87	**0.003**	11.93 ±2.00	12.12 ±1.45	0.736	10.70 ±1.94	13.00 ±1.41	0.101

Table S6. The association between first time participation and **all scores** of MMHLQ divided by **types** of PSGs.

	Autoimmune disease			Malignancy			Chronic disease			Genetic disease			Degenerative disease		
	1st time	Not 1st time	*p* value	1st time	Not 1st time	*p* value	1st time	Not 1st time	*p* value	1st time	Not 1st time	*p* value	1st time	Not 1st time	*p* value
**Accessing**															
1.Searching information about disease	2.81 ±0.60	2.88 ±0.67	0.666	2.65 ±0.55	2.33 ±0.78	0.147	2.81 ±0.79	2.89 ±0.64	0.681	2.85 ±0.67	2.94 ±0.44	0.513	2.71 ±0.62	3.00 ±1.41	0.822
2.Get information about health protection	2.84 ±0.52	2.96 ±0.61	0.427	2.84 ±0.37	2.58 ±0.51	0.137	2.91 ±0.68	2.89 ±0.64	0.912	2.94 ±0.56	3.00 ±0.37	0.675	2.83 ±0.61	3.50 ±0.71	0.128
3.Find health information from network	3.13 ±0.67	2.96 ±0.54	0.311	2.94 ±0.44	2.42 ±0.79	0.051	2.81 ±0.85	2.67 ±0.73	0.461	2.91 ±0.68	3.06 ±0.44	0.389	2.79 ±0.72	3.50 ±0.71	0.173
4. Get information about report of health examination report	2.74 ±0.58	2.68 ±0.48	0.668	2.77 ±0.43	2.42 ±0.79	0.161	2.88 ±0.70	2.85 ±0.53	0.840	2.89 ±0.62	3.00 ±0.52	0.521	2.70 ±0.60	3.50 ±0.71	0.069
Total	11.52 ±1.77	11.48 ±1.92	0.942	11.19 ±1.35	9.75 ±2.56	0.086	11.42 ±2.77	11.30 ±2.32	0.849	11.58 ±2.30	12.00 ±1.59	0.497	11.03 ±2.09	13.50 ±3.54	0.106
**Understanding**															
5. Understand the instruction of medication bag	2.97 ±0.66	3.04 ±0.54	0.660	3.00 ±0.37	2.83 ±0.58	0.367	3.00 ±0.58	3.04 ±0.44	0.776	3.03 ±0.64	3.00 ±0.52	0.858	2.94 ±0.54	3.50 ±0.71	0.148
6. Obey the instruction of medical personnel to care disease	3.10 ±0.65	3.08 ±0.70	0.927	2.94 ±0.51	2.67 ±0.49	0.127	3.02 ±0.64	3.04 ±0.52	0.925	3.05 ±0.57	3.00 ±0.63	0.777	3.05 ±0.50	3.50 ±0.71	0.216
7. Understand the introduction of medical personnel	3.10 ±0.47	3.16 ±0.47	0.621	2.94 ±0.44	2.83 ±0.39	0.487	3.02 ±0.64	3.04 ±0.44	0.922	3.05 ±0.51	3.06 ±0.57	0.912	3.06 ±0.49	3.50 ±0.71	0.216
8. Follow the instruction of medical bag to take medication	3.00 ±0.64	3.20 ±0.50	0.211	3.13 ±0.34	2.92 ±0.29	0.064	3.02 ±0.67	3.11 ±0.42	0.546	3.06 ±0.56	3.19 ±0.66	0.435	3.13 ±0.58	2.50 ±0.71	0.139
Total	12.26 ±1.95	12.48 ±1.92	0.671	12.00 ±1.06	11.25 ±1.42	0.117	12.07 ±2.19	12.22 ±1.72	0.759	12.18 ±2.10	12.25 ±2.05	0.911	12.18 ±1.67	13.00 ±2.83	0.498

Table S6. The association between first time participation and **all scores** of MMHLQ divided by **types** of PSGs. (Cont.)

	Autoimmune disease			Malignancy			Chronic disease			Genetic disease			Degenerative disease		
	1st time	Not 1st time	*p* value	1st time	Not 1st time	*p* value	1st time	Not 1st time	*p* value	1st time	Not 1st time	*p* value	1st time	Not 1st time	*p* value
**Evaluation**															
9. Evaluate whether the health information can be used to solve medical problems	2.74 ±0.63	2.68 ±0.48	0.686	2.65 ±0.49	2.42 ±0.51	0.181	2.81 ±0.63	2.74 ±0.59	0.629	2.71 ±0.70	2.75 ±0.58	0.824	2.54 ±0.64	2.50 ±0.71	0.935
10. Evaluate the health information suitable for himself/herself or not	2.71 ±0.64	2.60 ±0.58	0.505	2.65 ±0.55	2.50 ±0.52	0.436	2.79 ±0.64	2.67 ±0.68	0.443	2.77 ±0.63	2.81 ±0.54	0.802	2.56 ±0.59	2.50 ±0.71	0.883
11. Evaluate the difference or consistence of health information	2.35 ±0.71	2.56 ±0.51	0.213	2.71 ±0.53	2.50 ±0.52	0.249	2.77 ±0.68	2.59 ±0.69	0.304	2.72 ±0.70	2.88 ±0.62	0.427	2.45 ±0.61	3.00 ±0.00	**<0.001**
12. Evaluate the reliability of medical information from network	2.45 ±0.72	2.52 ±0.59	0.704	2.68 ±0.54	2.42 ±0.79	0.311	2.47 ±0.77	2.56 ±0.70	0.621	2.63 ±0.67	2.94 ±0.68	0.108	2.28 ±0.66	3.00 ±0.00	**<0.001**
Total	10.26 ±2.18	10.36 ±1.89	0.854	10.68 ±1.68	9.83 ±2.17	0.242	10.84 ±2.39	10.56 ±2.44	0.635	10.83 ±2.50	11.38 ±1.96	0.420	9.83 ±2.09	11.00 ±1.41	0.432
**Application**															
13. Apply health information to know the progress of disease	2.68 ±0.65	2.68 ±0.56	0.988	2.74 ±0.51	2.33 ±0.78	0.114	2.79 ±0.56	2.85 ±0.53	0.652	2.77 ±0.68	2.81 ±0.40	0.808	2.55 ±0.53	3.00 ±0.00	**<0.001**
14. Apply health information to prevent disease	2.87 ±0.50	2.76 ±0.60	0.452	2.71 ±0.53	2.42 ±0.51	0.108	2.79 ±0.60	2.89 ±0.42	0.461	2.82 ±0.63	2.88 ±0.62	0.736	2.65 ±0.58	3.00 ±0.00	**<0.001**
15. Apply health information to understand the report of health examination	2.94 ±0.51	2.72 ±0.61	0.167	2.77 ±0.43	2.50 ±0.80	0.278	2.86 ±0.52	2.96 ±0.34	0.318	2.89 ±0.62	2.88 ±0.50	0.917	2.73 ±0.59	3.00 ±0.00	**<0.001**
16. Apply health information to decided how treat disease	2.77 ±0.62	2.72 ±0.61	0.745	2.58 ±0.50	2.33 ±0.65	0.190	2.88 ±0.50	2.78 ±0.58	0.418	2.77 ±0.66	2.75 ±0.45	0.912	2.59 ±0.54	3.00 ±0.00	**<0.001**
Total	11.26 ±1.61	10.88 ±2.11	0.450	10.81 ±1.49	9.58 ±2.35	0.115	11.33 ±1.98	11.48 ±1.50	0.728	11.25 ±2.47	11.31 ±1.70	0.919	10.51 ±1.88	12.00 ±0.00	**<0.001**

Table S6. The association between first time participation and **all scores** of MMHLQ divided by **types** of PSGs. (Cont.)

	Autoimmune disease			Malignancy			Chronic disease			Genetic disease			Degenerative disease		
	1st time	Not 1st time	*p* value	1st time	Not 1st time	*p* value	1st time	Not 1st time	*p* value	1st time	Not 1st time	*p* value	1st time	Not 1st time	*p* value
**Communication**															
17. Talk to doctors the chosen examination and treatment	2.84 ±0.64	2.88 ±0.73	0.822	2.81 ±0.54	2.58 ±0.51	0.227	2.79 ±0.56	2.81 ±0.62	0.867	2.94 ±0.58	2.94 ±0.44	0.995	2.55 ±0.61	3.00 ±0.00	**<0.001**
18. Make sure with medical personnel about accuracy of orders	3.03 ±0.48	2.88 ±0.44	0.227	2.90 ±0.60	2.42 ±0.79	0.072	2.86 ±0.52	2.85 ±0.46	0.944	2.91 ±0.61	2.88 ±0.50	0.842	2.73 ±0.59	3.00 ±0.00	0.518
19. Discuss with doctor about the choice of treatment	3.03 ±0.55	3.08 ±0.49	0.736	2.87 ±0.62	2.83 ±0.58	0.856	2.81 ±0.50	2.96 ±0.52	0.235	2.89 ±0.62	3.06 ±0.57	0.319	2.70 ±0.56	3.50 ±0.71	0.050
20. Ask medical personnel if you are not sure	2.94 ±0.36	3.04 ±0.35	0.279	2.90 ±0.54	2.67 ±0.65	0.230	2.79 ±0.56	3.00 ±0.48	0.113	2.94 ±0.58	3.06 ±0.57	0.447	2.73 ±0.57	3.50 ±0.71	0.063
Total	11.84 ±1.71	11.88 ±1.69	0.929	11.48 ±2.01	10.50 ±2.07	0.161	11.26 ±1.83	11.63 ±1.82	0.407	11.68 ±2.21	11.94 ±1.95	0.667	10.70 ±1.94	13.00 ±1.41	0.101

Table S7. The association between participation (patient or family) and **all scores** of MMHLQ divided by **PSGs**

	SLE			Head-and-neck cancer			DM			ADPKD			Hemodialysis			CKD			COPD			Osteoporosis		
	Not family	family	*p* value	Not family	family	*p* value	Not family	family	*p* value	Not family	family	*p* value	Not family	family	*p* value	Not family	family	*p* value	Not family	family	*p* value	Not family	family	*p* value
**Accessing**																								
1.Searching information about disease	3.00 ±0.55	2.59 ±0.67	**0.021**	2.52 ±0.70	2.63 ±0.50	0.597	2.83 ±0.77	2.88 ±0.62	0.844	3.00 ±0.68	2.80 ±0.59	0.170	2.72 ±0.54	2.94 ±0.42	0.132	2.65 ±0.93	3.33 ±0.59	**0.012**	2.71 ±0.72	2.64 ±0.50	0.768	2.71 ±0.64	2.74 ±0.62	0.862
2.Get information about health protection	2.97 ±0.58	2.77 ±0.53	0.201	2.78 ±0.42	2.75 ±0.45	0.840	2.91 ±0.68	2.88 ±0.62	0.865	3.07 ±0.47	2.89 ±0.54	0.133	2.76 ±0.52	3.06 ±0.42	**0.046**	2.70 ±0.86	3.39 ±0.50	**0.005**	2.91 ±0.71	3.00 ±0.45	0.702	2.83 ±0.62	2.87 ±0.63	0.799
3.Find health information from network	3.09 ±0.67	3.00 ±0.53	0.605	2.78 ±0.64	2.81 ±0.54	0.857	2.78 ±0.82	2.69 ±0.79	0.697	3.04 ±0.71	2.89 ±0.60	0.329	2.72 ±0.79	3.00 ±0.49	0.159	2.70 ±0.92	3.39 ±0.61	**0.011**	2.76 ±0.78	2.91 ±0.30	0.377	2.75 ±0.80	2.96 ±0.47	0.147
4. Get information about report of health examination report	2.76 ±0.50	2.64 ±0.58	0.381	2.67 ±0.55	2.69 ±0.60	0.909	2.93 ±0.61	2.69 ±0.70	0.189	3.07 ±0.55	2.83 ±0.61	0.086	2.60 ±0.50	2.89 ±0.58	0.099	2.80 ±0.89	3.44 ±0.51	**0.011**	2.88 ±0.69	2.64 ±0.50	0.280	2.69 ±0.65	2.78 ±0.52	0.565
Total	11.82 ±1.87	11.00 ±1.66	0.098	10.74 ±1.95	10.88 ±1.75	0.822	11.44 ±2.63	11.13 ±2.50	0.668	12.19 ±2.08	11.41 ±2.19	0.130	10.80 ±1.63	11.89 ±1.45	**0.029**	10.85 ±3.45	13.56 ±2.04	**0.006**	11.26 ±2.40	11.18 ±1.47	0.915	10.98 ±2.25	11.35 ±1.85	0.491

Table S7. The association between participation (patient or family) and **all scores** of MMHLQ divided by **PSGs** (Cont.)

	SLE			Head-and-neck cancer			DM			ADPKD			Hemodialysis			CKD			COPD			Osteoporosis		
	Not family	family	*p* value	Not family	family	*p* value	Not family	family	*p* value	Not family	family	*p* value	Not family	family	*p* value	Not family	family	*p* value	Not family	family	*p* value	Not family	family	*p* value
**Understanding**																								
5. Understand the instruction of medication bag	3.09 ±0.62	2.86 ±0.56	0.176	3.00 ±0.39	2.88 ±0.50	0.367	3.04 ±0.55	2.94 ±0.44	0.509	3.22 ±0.70	2.93 ±0.54	**0.039**	3.04 ±0.35	3.00 ±0.49	0.755	2.95 ±0.69	3.33 ±0.59	0.075	3.18 ±0.52	2.82 ±0.75	0.083	2.97 ±0.52	2.91 ±0.60	0.693
6. Obey the instruction of medical personnel to care disease	3.18 ±0.72	2.95 ±0.58	0.207	2.89 ±0.42	2.81 ±0.66	0.680	3.06 ±0.66	2.94 ±0.25	0.283	3.26 ±0.66	2.93 ±0.51	**0.014**	3.04 ±0.45	2.94 ±0.64	0.569	2.95 ±0.69	3.33 ±0.69	0.094	3.21 ±0.41	2.91 ±0.54	0.060	3.10 ±0.48	2.96 ±0.56	0.245
7. Understand the introduction of medical personnel	3.15 ±0.56	3.09 ±0.29	0.626	2.96 ±0.44	2.81 ±0.40	0.268	3.04 ±0.61	3.00 ±0.37	0.819	3.22 ±0.51	2.96 ±0.51	**0.034**	3.00 ±0.41	3.11 ±0.47	0.414	2.90 ±0.72	3.39 ±0.50	**0.021**	3.21 ±0.48	3.18 ±0.40	0.881	3.10 ±0.48	3.00 ±0.52	0.403
8. Follow the instruction of medical bag to take medication	3.15 ±0.56	3.00 ±0.63	0.371	3.15 ±0.36	2.94 ±0.25	0.047	3.06 ±0.66	3.06 ±0.25	0.949	3.33 ±0.55	2.96 ±0.55	**0.005**	3.16 ±0.47	3.17 ±0.38	0.961	3.05 ±0.51	3.39 ±0.50	**0.047**	3.26 ±0.51	3.00 ±0.45	0.116	3.12 ±0.59	3.09 ±0.60	0.828
Total	12.56 ±2.19	12.05 ±1.40	0.333	12.00 ±1.04	11.44 ±1.41	0.141	12.19 ±2.21	11.94 ±1.12	0.550	13.04 ±2.17	11.78 ±1.91	**0.009**	12.24 ±1.33	12.22 ±1.63	0.969	11.85 ±2.41	13.44 ±2.18	**0.040**	12.85 ±1.65	11.91 ±1.76	0.112	12.29 ±1.58	11.96 ±1.97	0.428

Table S7. The association between participation (patient or family) and **all scores** of MMHLQ divided by **PSGs** (Cont.)

	SLE			Head-and-neck cancer			DM			ADPKD			Hemodialysis			CKD			COPD			Osteoporosis		
	Not family	family	*p* value	Not family	family	*p* value	Not family	family	*p* value	Not family	family	*p* value	Not family	family	*p* value	Not family	family	*p* value	Not family	family	*p* value	Not family	family	*p* value
**Evaluation**																								
9. Evaluate whether the health information can be used to solve medical problems	2.79 ±0.54	2.59 ±0.59	0.190	2.63 ±0.49	2.50 ±0.52	0.417	2.83 ±0.61	2.63 ±0.62	0.234	2.93 ±0.73	2.61 ±0.63	**0.047**	2.64 ±0.49	2.78 ±0.73	0.463	2.65 ±0.75	3.28 ±0.67	**0.010**	2.94 ±0.55	2.73 ±0.65	0.287	2.51 ±0.68	2.61 ±0.50	0.522
10. Evaluate the health information suitable for himself/herself or not	2.76 ±0.61	2.50 ±0.60	0.114	2.70 ±0.54	2.44 ±0.51	0.120	2.74 ±0.68	2.75 ±0.58	0.961	2.96 ±0.65	2.69 ±0.58	0.054	2.68 ±0.63	2.83 ±0.71	0.458	2.80 ±0.70	3.28 ±0.67	**0.038**	2.97 ±0.58	2.64 ±0.50	0.093	2.59 ±0.62	2.48 ±0.51	0.431
11. Evaluate the difference or consistence of health information	2.56 ±0.66	2.27 ±0.55	0.097	2.70 ±0.54	2.56 ±0.51	0.404	2.69 ±0.72	2.75 ±0.58	0.743	2.96 ±0.76	2.65 ±0.62	**0.049**	2.64 ±0.64	2.61 ±0.61	0.882	2.55 ±0.83	3.28 ±0.67	**0.005**	2.91 ±0.67	2.73 ±0.47	0.401	2.46 ±0.60	2.48 ±0.67	0.892
12. Evaluate the reliability of medical information from network	2.59 ±0.66	2.32 ±0.65	0.136	2.67 ±0.62	2.50 ±0.63	0.403	2.48 ±0.75	2.56 ±0.73	0.702	2.85 ±0.72	2.61 ±0.66	0.136	2.56 ±0.71	2.61 ±0.78	0.824	2.45 ±0.89	3.28 ±0.67	**0.003**	2.68 ±0.73	2.64 ±0.50	0.866	2.22 ±0.65	2.48 ±0.67	0.111
Total	10.71 ±2.07	9.68 ±1.86	0.065	10.70 ±1.81	10.00 ±1.86	0.230	10.74 ±2.44	10.69 ±2.30	0.938	11.70 ±2.69	10.56 ±2.17	**0.042**	10.52 ±2.08	10.83 ±2.53	0.659	10.45 ±2.91	13.11 ±2.68	**0.006**	11.50 ±2.26	10.73 ±2.00	0.318	9.78 ±2.11	10.04 ±2.01	0.608

Table S7. The association between participation (patient or family) and **all scores** of MMHLQ divided by **PSGs** (Cont.)

	SLE			Head-and-neck cancer			DM			ADPKD			Hemodialysis			CKD			COPD			Osteoporosis		
	Not family	family	*p* value	Not family	family	*p* value	Not family	family	*p* value	Not family	family	*p* value	Not family	family	*p* value	Not family	family	*p* value	Not family	family	*p* value	Not family	family	*p* value
**Application**																								
13. Apply health information to know the progress of disease	2.79 ±0.59	2.50 ±0.60	0.076	2.70 ±0.61	2.50 ±0.63	0.302	2.80 ±0.56	2.88 ±0.50	0.616	2.96 ±0.71	2.69 ±0.58	0.062	2.80 ±0.50	2.67 ±0.59	0.430	2.85 ±0.81	3.33 ±0.59	**0.045**	2.82 ±0.63	2.73 ±0.65	0.662	2.53 ±0.54	2.65 ±0.49	0.328
14. Apply health information to prevent disease	2.94 ±0.55	2.64 ±0.49	**0.039**	2.67 ±0.48	2.56 ±0.63	0.544	2.80 ±0.56	2.94 ±0.44	0.360	3.00 ±0.73	2.74 ±0.56	0.080	2.84 ±0.37	2.83 ±0.38	0.955	2.85 ±0.67	3.33 ±0.59	**0.025**	2.97 ±0.63	2.82 ±0.40	0.455	2.61 ±0.62	2.78 ±0.42	0.153
15. Apply health information to understand the report of health examination	2.91 ±0.57	2.73 ±0.55	0.236	2.70 ±0.54	2.69 ±0.60	0.928	2.89 ±0.46	2.94 ±0.44	0.710	3.11 ±0.64	2.78 ±0.54	**0.016**	3.00 ±0.29	2.83 ±0.51	0.226	2.95 ±0.60	3.39 ±0.50	**0.021**	2.82 ±0.63	2.73 ±0.47	0.642	2.71 ±0.62	2.78 ±0.52	0.628
16. Apply health information to decided how treat disease	2.97 ±0.52	2.41 ±0.59	**0.001**	2.56 ±0.58	2.44 ±0.51	0.504	2.85 ±0.53	2.81 ±0.54	0.796	2.93 ±0.73	2.69 ±0.54	0.099	2.84 ±0.37	2.72 ±0.57	0.453	2.90 ±0.64	3.28 ±0.67	0.084	2.85 ±0.61	2.73 ±0.65	0.561	2.54 ±0.54	2.74 ±0.54	0.140
Total	11.62 ±1.83	10.27 ±1.58	**0.006**	10.63 ±1.80	10.19 ±1.91	0.451	11.33 ±1.85	11.56 ±1.67	0.659	12.00 ±2.72	10.89 ±2.03	**0.042**	11.48 ±1.16	11.06 ±1.55	0.310	11.55 ±2.52	13.33 ±2.25	**0.028**	11.47 ±2.27	11.00 ±1.84	0.537	10.39 ±1.91	10.96 ±1.74	0.221

Table S7. The association between participation (patient or family) and **all scores** of MMHLQ divided by **PSGs** (Cont.)

	SLE			Head-and-neck cancer			DM			ADPKD			Hemodialysis			CKD			COPD			Osteoporosis		
	Not family	family	*p* value	Not family	family	*p* value	Not family	family	*p* value	Not family	family	*p* value	Not family	family	*p* value	Not family	family	*p* value	Not family	family	*p* value	Not family	family	*p* value
**Communication**																								
17. Talk to doctors the chosen examination and treatment	2.97 ±0.72	2.68 ±0.57	0.117	2.81 ±0.48	2.63 ±0.62	0.269	2.81 ±0.59	2.75 ±0.58	0.698	3.04 ±0.59	2.89 ±0.54	0.260	2.80 ±0.41	2.83 ±0.51	0.814	3.10 ±0.55	3.33 ±0.69	0.254	2.91 ±0.62	2.91 ±0.30	0.989	2.58 ±0.59	2.52 ±0.67	0.719
18. Make sure with medical personnel about accuracy of orders	2.97 ±0.46	2.95 ±0.49	0.901	2.85 ±0.60	2.63 ±0.81	0.299	2.85 ±0.53	2.88 ±0.34	0.870	3.04 ±0.65	2.83 ±0.54	0.139	2.92 ±0.40	2.94 ±0.54	0.865	3.15 ±0.49	3.33 ±0.59	0.304	2.94 ±0.55	2.91 ±0.30	0.854	2.71 ±0.62	2.78 ±0.52	0.628
19. Discuss with doctor about the choice of treatment	3.09 ±0.57	3.00 ±0.44	0.540	2.96 ±0.59	2.69 ±0.60	0.148	2.87 ±0.55	2.88 ±0.34	0.975	3.11 ±0.70	2.83 ±0.54	0.052	2.88 ±0.33	2.89 ±0.68	0.959	2.95 ±0.69	3.28 ±0.67	0.146	3.12 ±0.48	3.09 ±0.54	0.876	2.69 ±0.59	2.78 ±0.52	0.537
20. Ask medical personnel if you are not sure	3.03 ±0.39	2.91 ±0.29	0.220	2.89 ±0.58	2.75 ±0.58	0.450	2.87 ±0.58	2.88 ±0.34	0.976	3.22 ±0.51	2.83 ±0.57	**0.004**	2.96 ±0.35	2.94 ±0.54	0.909	3.10 ±0.55	3.33 ±0.59	0.218	3.03 ±0.58	3.09 ±0.54	0.757	2.76 ±0.57	2.70 ±0.63	0.643
Total	12.06 ±1.84	11.55 ±1.41	0.270	11.52 ±1.93	10.69 ±2.21	0.203	11.41 ±1.96	11.38 ±1.31	0.951	12.41 ±2.22	11.39 ±2.05	**0.044**	11.56 ±1.12	11.61 ±2.00	0.916	12.30 ±2.13	13.28 ±2.47	0.198	12.00 ±1.91	12.00 ±1.48	1.000	10.75 ±1.94	10.78 ±2.04	0.940

Table S8. The association between participation (patient or family) and **all scores** of MMHLQ divided by **types** of PSGs

	Autoimmune disease			Malignancy			Chronic disease			Genetic disease			Degenerative disease		
	Not family	family	*p* value	Not family	family	*p* value	Not family	family	*p* value	Not family	family	*p* value	Not family	family	*p* value
**Accessing**															
1.Searching information about disease	3.00 ±0.55	2.59 ±0.67	**0.021**	2.52 ±0.70	2.63 ±0.50	0.597	2.83 ±0.77	2.88 ±0.62	0.844	3.00 ±0.68	2.80 ±0.59	0.170	2.71 ±0.64	2.74 ±0.62	0.862
2.Get information about health protection	2.97 ±0.58	2.77 ±0.53	0.201	2.78 ±0.42	2.75 ±0.45	0.840	2.91 ±0.68	2.88 ±0.62	0.865	3.07 ±0.47	2.89 ±0.54	0.133	2.83 ±0.62	2.87 ±0.63	0.799
3.Find health information from network	3.09 ±0.67	3.00 ±0.53	0.605	2.78 ±0.64	2.81 ±0.54	0.857	2.78 ±0.82	2.69 ±0.79	0.697	3.04 ±0.71	2.89 ±0.60	0.329	2.75 ±0.80	2.96 ±0.47	0.147
4. Get information about report of health examination report	2.76 ±0.50	2.64 ±0.58	0.381	2.67 ±0.55	2.69 ±0.60	0.909	2.93 ±0.61	2.69 ±0.70	0.189	3.07 ±0.55	2.83 ±0.61	0.086	2.69 ±0.65	2.78 ±0.52	0.565
Total	11.82 ±1.87	11.00 ±1.66	0.098	10.74 ±1.95	10.88 ±1.75	0.822	11.44 ±2.63	11.13 ±2.50	0.668	12.19 ±2.08	11.41 ±2.19	0.130	10.98 ±2.25	11.35 ±1.85	0.491
**Understanding**															
5. Understand the instruction of medication bag	3.09 ±0.62	2.86 ±0.56	0.176	3.00 ±0.39	2.88 ±0.50	0.367	3.04 ±0.55	2.94 ±0.44	0.509	3.22 ±0.70	2.93 ±0.54	**0.039**	2.97 ±0.52	2.91 ±0.60	0.693
6. Obey the instruction of medical personnel to care disease	3.18 ±0.72	2.95 ±0.58	0.207	2.89 ±0.42	2.81 ±0.66	0.680	3.06 ±0.66	2.94 ±0.25	0.283	3.26 ±0.66	2.93 ±0.51	**0.014**	3.10 ±0.48	2.96 ±0.56	0.245
7. Understand the introduction of medical personnel	3.15 ±0.56	3.09 ±0.29	0.626	2.96 ±0.44	2.81 ±0.40	0.268	3.04 ±0.61	3.00 ±0.37	0.819	3.22 ±0.51	2.96 ±0.51	**0.034**	3.10 ±0.48	3.00 ±0.52	0.403
8. Follow the instruction of medical bag to take medication	3.15 ±0.56	3.00 ±0.63	0.371	3.15 ±0.36	2.94 ±0.25	0.047	3.06 ±0.66	3.06 ±0.25	0.967	3.33 ±0.55	2.96 ±0.55	**0.005**	3.12 ±0.59	3.09 ±0.60	0.828
Total	12.56 ±2.19	12.05 ±1.40	0.333	12.00 ±1.04	11.44 ±1.41	0.141	12.19 ±2.21	11.94 ±1.12	0.550	13.04 ±2.17	11.78 ±1.91	**0.009**	12.29 ±1.58	11.96 ±1.97	0.428

Table S8. The association between participation (patient or family) and **all scores** of MMHLQ divided by **types** of PSGs (Cont.)

	Autoimmune disease			Malignancy			Chronic disease			Genetic disease			Degenerative disease		
	Not family	family	*p* value	Not family	family	*p* value	Not family	family	*p* value	Not family	family	*p* value	Not family	family	*p* value
**Evaluation**															
9. Evaluate whether the health information can be used to solve medical problems	2.79 ±0.54	2.59 ±0.59	0.190	2.63 ±0.49	2.50 ±0.52	0.417	2.83 ±0.61	2.63 ±0.62	0.234	2.93 ±0.73	2.61 ±0.63	**0.047**	2.51 ±0.68	2.61 ±0.50	0.522
10. Evaluate the health information suitable for himself/herself or not	2.76 ±0.61	2.50 ±0.60	0.114	2.70 ±0.54	2.44 ±0.51	0.120	2.74 ±0.68	2.75 ±0.58	0.961	2.96 ±0.65	2.69 ±0.58	0.054	2.59 ±0.62	2.48 ±0.51	0.431
11. Evaluate the difference or consistence of health information	2.56 ±0.66	2.27 ±0.55	0.097	2.70 ±0.54	2.56 ±0.51	0.404	2.69 ±0.72	2.75 ±0.58	0.743	2.96 ±0.76	2.65 ±0.62	**0.049**	2.46 ±0.60	2.48 ±0.67	0.892
12. Evaluate the reliability of medical information from network	2.59 ±0.66	2.32 ±0.65	0.136	2.67 ±0.62	2.50 ±0.63	0.403	2.48 ±0.75	2.56 ±0.73	0.702	2.85 ±0.72	2.61 ±0.66	0.136	2.22 ±0.65	2.48 ±0.67	0.111
Total	10.71 ±2.07	9.68 ±1.86	0.065	10.70 ±1.81	10.00 ±1.86	0.230	10.74 ±2.44	10.69 ±2.30	0.938	11.70 ±2.69	10.56 ±2.17	**0.042**	9.78 ±2.11	10.04 ±2.01	0.608
**Application**															
13. Apply health information to know the progress of disease	2.79 ±0.59	2.50 ±0.60	0.076	2.70 ±0.61	2.50 ±0.63	0.302	2.80 ±0.56	2.88 ±0.50	0.616	2.96 ±0.71	2.69 ±0.58	0.062	2.53 ±0.54	2.65 ±0.49	0.328
14. Apply health information to prevent disease	2.94 ±0.55	2.64 ±0.49	**0.039**	2.67 ±0.48	2.56 ±0.63	0.544	2.80 ±0.56	2.94 ±0.44	0.360	3.00 ±0.73	2.74 ±0.56	0.080	2.61 ±0.62	2.78 ±0.42	0.153
15. Apply health information to understand the report of health examination	2.91 ±0.57	2.73 ±0.55	0.236	2.70 ±0.54	2.69 ±0.60	0.928	2.89 ±0.46	2.94 ±0.44	0.710	3.11 ±0.64	2.78 ±0.54	**0.016**	2.71 ±0.62	2.78 ±0.52	0.628
16. Apply health information to decided how treat disease	2.97 ±0.52	2.41 ±0.59	**0.001**	2.56 ±0.58	2.44 ±0.51	0.504	2.85 ±0.53	2.81 ±0.54	0.796	2.93 ±0.73	2.69 ±0.54	0.099	2.54 ±0.54	2.74 ±0.54	0.140
Total	11.62 ±1.83	10.27 ±1.58	**0.006**	10.63 ±1.80	10.19 ±1.91	0.451	11.33 ±1.85	11.56 ±1.67	0.659	12.00 ±2.72	10.89 ±2.03	**0.042**	10.39 ±1.91	10.96 ±1.74	0.221

Table S8. The association between participation (patient or family) and **all scores** of MMHLQ divided by **types** of PSGs (Cont.)

	Autoimmune disease			Malignancy			Chronic disease			Genetic disease			Degenerative disease		
	Not family	family	*p* value	Not family	family	*p* value	Not family	family	*p* value	Not family	family	*p* value	Not family	family	*p* value
**Communication**															
17. Talk to doctors the chosen examination and treatment	2.97 ±0.72	2.68 ±0.57	0.117	2.81 ±0.48	2.63 ±0.62	0.269	2.81 ±0.59	2.75 ±0.58	0.698	3.04 ±0.59	2.89 ±0.54	0.260	2.58 ±0.59	2.52 ±0.67	0.719
18. Make sure with medical personnel about accuracy of orders	2.97 ±0.46	2.95 ±0.49	0.901	2.85 ±0.60	2.63 ±0.81	0.299	2.85 ±0.53	2.88 ±0.34	0.870	3.04 ±0.65	2.83 ±0.54	0.139	2.71 ±0.62	2.78 ±0.52	0.628
19. Discuss with doctor about the choice of treatment	3.09 ±0.57	3.00 ±0.44	0.540	2.96 ±0.59	2.69 ±0.60	0.148	2.87 ±0.55	2.88 ±0.34	0.975	3.11 ±0.70	2.83 ±0.54	0.052	2.69 ±0.59	2.78 ±0.52	0.537
20. Ask medical personnel if you are not sure	3.03 ±0.39	2.91 ±0.29	0.220	2.89 ±0.58	2.75 ±0.58	0.450	2.87 ±0.58	2.88 ±0.34	0.976	3.22 ±0.51	2.83 ±0.57	**0.004**	2.76 ±0.57	2.70 ±0.63	0.643
Total	12.06 ±1.84	11.55 ±1.41	0.270	11.52 ±1.93	10.69 ±2.21	0.203	11.41 ±1.96	11.38 ±1.31	0.951	12.41 ±2.22	11.39 ±2.05	**0.044**	10.75 ±1.94	10.78 ±2.04	0.940

Table S9. The association between age (<65y/o or ≥65 y/o) and **all scores** of MMHLQ divided by **PSGs**

	SLE			Head-and-neck cancer			DM			ADPKD			Hemodialysis			CKD			COPD			Osteoporosis		
	Age<65	Age≥ 65	*p* value	Age<65	Age≥ 65	*p* value	Age<65	Age≥ 65	*p* value	Age<65	Age≥ 65	*p* value	Age<65	Age≥ 65	*p* value	Age<65	Age≥ 65	*p* value	Age<65	Age≥ 65	*p* value	Age<65	Age≥ 65	*p* value
**Accessing**																								
1.Searching information about disease	2.88 ±0.63	2.57 ±0.53	0.229	2.58 ±0.65	2.43 ±0.53	0.558	3.03 ±0.63	2.61 ±0.80	**0.022**	2.88 ±0.60	2.81 ±0.75	0.715	2.78 ±0.48	3.00 ±0.63	0.332	3.21 ±0.74	2.30 ±0.82	**0.002**	2.94 ±0.64	2.52 ±0.64	**0.035**	2.85 ±0.63	2.60 ±0.62	0.085
2.Get information about health protection	2.92 ±0.57	2.71 ±0.49	0.373	2.78 ±0.42	2.71 ±0.49	0.724	3.10 ±0.55	2.65 ±0.71	**0.005**	2.95 ±0.48	2.94 ±0.68	0.912	2.86 ±0.48	3.00 ±0.63	0.544	3.21 ±0.74	2.50 ±0.71	**0.012**	3.06 ±0.64	2.85 ±0.66	0.311	2.97 ±0.58	2.72 ±0.63	0.062
3.Find health information from network	3.10 ±0.62	2.71 ±0.49	0.120	2.81 ±0.62	2.71 ±0.49	0.717	3.00 ±0.73	2.45 ±0.81	**0.004**	3.00 ±0.53	2.69 ±0.95	0.220	2.97 ±0.50	2.00 ±1.10	0.082	3.25 ±0.80	2.40 ±0.70	**0.005**	3.11 ±0.68	2.59 ±0.64	**0.012**	3.05 ±0.51	2.58 ±0.82	**0.002**
4. Get information about report of health examination report	2.73 ±0.53	2.57 ±0.53	0.451	2.72 ±0.57	2.43 ±0.53	0.213	3.05 ±0.56	2.65 ±0.66	**0.008**	2.94 ±0.53	2.81 ±0.83	0.571	2.70 ±0.57	2.83 ±0.41	0.595	3.21 ±0.74	2.80 ±0.92	0.162	3.11 ±0.68	2.63 ±0.56	**0.013**	2.77 ±0.58	2.67 ±0.64	0.488
Total	11.63 ±1.84	10.57 ±1.40	0.150	10.89 ±1.89	10.29 ±1.70	0.439	12.18 ±2.21	10.35 ±2.70	**0.003**	11.77 ±1.91	11.25 ±3.07	0.526	11.32 ±1.60	10.83 ±1.94	0.501	12.89 ±2.96	10.00 ±2.75	**0.011**	12.22 ±2.16	10.59 ±2.00	**0.013**	11.64 ±1.90	10.58 ±2.24	**0.024**
**Understanding**																								
5. Understand the instruction of medication bag	3.02 ±0.63	2.86 ±0.38	0.508	2.97 ±0.45	2.86 ±0.38	0.527	3.13 ±0.47	2.87 ±0.56	**0.041**	3.06 ±0.53	2.88 ±0.89	0.430	3.00 ±0.41	3.17 ±0.41	0.359	3.29 ±0.60	2.70 ±0.67	**0.015**	3.22 ±0.65	3.00 ±0.55	0.225	2.95 ±0.65	2.95 ±0.43	0.969
6. Obey the instruction of medical personnel to care disease	3.08 ±0.67	3.14 ±0.69	0.823	2.92 ±0.50	2.57 ±0.53	0.106	3.15 ±0.49	2.87 ±0.67	**0.045**	3.06 ±0.50	2.94 ±0.85	0.584	2.97 ±0.55	3.17 ±0.41	0.417	3.25 ±0.65	2.80 ±0.79	0.083	3.39 ±0.50	2.96 ±0.34	**0.004**	3.00 ±0.56	3.12 ±0.45	0.301
7. Understand the introduction of medical personnel	3.14 ±0.46	3.00 ±0.58	0.457	2.94 ±0.41	2.71 ±0.49	0.195	3.21 ±0.41	2.81 ±0.65	**0.003**	3.09 ±0.46	2.88 ±0.72	0.264	3.05 ±0.47	3.00 ±0.00	0.781	3.29 ±0.60	2.70 ±0.67	**0.015**	3.44 ±0.51	3.04 ±0.34	**0.006**	3.08 ±0.53	3.07 ±0.46	0.948
8. Follow the instruction of medical bag to take medication	3.10 ±0.59	3.00 ±0.58	0.665	3.08 ±0.37	3.00 ±0.00	0.557	3.15 ±0.49	2.94 ±0.68	0.123	3.12 ±0.55	2.94 ±0.68	0.249	3.16 ±0.44	3.17 ±0.41	0.981	3.32 ±0.55	2.90 ±0.32	**0.007**	3.33 ±0.59	3.11 ±0.42	0.181	3.10 ±0.60	3.12 ±0.59	0.917
Total	12.41 ±1.91	12.00 ±2.08	0.603	11.92 ±1.20	11.14 ±1.07	0.122	12.64 ±1.60	11.48 ±2.29	**0.015**	12.34 ±1.81	11.63 ±2.92	0.362	12.19 ±1.49	12.50 ±1.22	0.631	13.14 ±2.27	11.10 ±2.23	**0.019**	13.39 ±2.06	12.11 ±1.22	**0.026**	12.13 ±1.88	12.26 ±1.51	0.735

Table S9. The association between age (<65y/o or ≥65 y/o) and **all scores** of MMHLQ divided by **PSGs** (Cont.)

	SLE			Head-and-neck cancer			DM			ADPKD			Hemodialysis			CKD			COPD			Osteoporosis		
	Age<65	Age≥ 65	*p* value	Age<65	Age≥ 65	*p* value	Age<65	Age≥ 65	*p* value	Age<65	Age≥ 65	*p* value	Age<65	Age≥ 65	*p* value	Age<65	Age≥ 65	*p* value	Age<65	Age≥ 65	*p* value	Age<65	Age≥ 65	*p* value
**Evaluation**																								
9. Evaluate whether the health information can be used to solve medical problems	2.69 ±0.58	2.86 ±0.38	0.346	2.64 ±0.49	2.29 ±0.49	0.087	2.95 ±0.56	2.58 ±0.62	**0.012**	2.72 ±0.65	2.69 ±0.79	0.852	2.73 ±0.61	2.50 ±0.55	0.390	3.14 ±0.76	2.40 ±0.52	**0.007**	3.00 ±0.49	2.81 ±0.62	0.270	2.62 ±0.63	2.47 ±0.63	0.285
10. Evaluate the health information suitable for himself/herself or not	2.65 ±0.63	2.71 ±0.49	0.807	2.64 ±0.54	2.43 ±0.53	0.353	2.90 ±0.60	2.55 ±0.68	**0.025**	2.78 ±0.57	2.75 ±0.77	0.841	2.78 ±0.63	2.50 ±0.84	0.333	3.18 ±0.72	2.60 ±0.52	**0.026**	3.00 ±0.59	2.81 ±0.56	0.293	2.59 ±0.59	2.53 ±0.59	0.677
11. Evaluate the difference or consistence of health information	2.45 ±0.65	2.43 ±0.53	0.937	2.69 ±0.52	2.43 ±0.53	0.228	2.82 ±0.60	2.55 ±0.77	0.111	2.75 ±0.64	2.75 ±0.86	0.984	2.68 ±0.58	2.33 ±0.82	0.212	3.11 ±0.79	2.30 ±0.67	**0.007**	3.06 ±0.54	2.74 ±0.66	0.098	2.49 ±0.68	2.44 ±0.55	0.740
12. Evaluate the reliability of medical information from network	2.51 ±0.68	2.29 ±0.49	0.405	2.58 ±0.65	2.71 ±0.49	0.616	2.62 ±0.71	2.35 ±0.75	0.143	2.68 ±0.64	2.75 ±0.86	0.704	2.62 ±0.76	2.33 ±0.52	0.377	3.07 ±0.86	2.20 ±0.63	**0.006**	2.83 ±0.71	2.56 ±0.64	0.179	2.38 ±0.71	2.21 ±0.60	0.230
Total	10.31 ±2.11	10.29 ±1.50	0.975	10.56 ±1.87	9.86 ±1.68	0.365	11.28 ±2.18	10.03 ±2.51	**0.029**	10.94 ±2.19	10.94 ±3.21	0.999	10.81 ±2.22	9.67 ±2.42	0.254	12.50 ±3.05	9.50 ±1.90	**0.006**	11.89 ±2.14	10.93 ±2.20	0.153	10.08 ±2.25	9.65 ±1.90	0.356
**Application**																								
13. Apply health information to know the progress of disease	2.71 ±0.61	2.43 ±0.53	0.247	2.69 ±0.58	2.29 ±0.76	0.110	2.97 ±0.43	2.61 ±0.62	**0.008**	2.78 ±0.57	2.75 ±0.86	0.880	2.76 ±0.55	2.67 ±0.52	0.709	3.29 ±0.66	2.50 ±0.71	**0.003**	3.00 ±0.59	2.67 ±0.62	0.080	2.62 ±0.54	2.51 ±0.51	0.373
14. Apply health information to prevent disease	2.84 ±0.55	2.71 ±0.49	0.581	2.69 ±0.47	2.29 ±0.76	0.210	2.97 ±0.36	2.65 ±0.66	**0.017**	2.85 ±0.57	2.75 ±0.86	0.674	2.84 ±0.37	2.83 ±0.41	0.979	3.21 ±0.69	2.70 ±0.48	**0.036**	3.17 ±0.38	2.78 ±0.64	**0.026**	2.77 ±0.54	2.56 ±0.59	0.095
15. Apply health information to understand the report of health examination	2.86 ±0.58	2.71 ±0.49	0.536	2.72 ±0.57	2.57 ±0.53	0.519	3.03 ±0.28	2.74 ±0.58	**0.016**	2.92 ±0.51	2.75 ±0.86	0.448	2.95 ±0.40	2.83 ±0.41	0.531	3.29 ±0.60	2.80 ±0.42	**0.024**	3.00 ±0.59	2.67 ±0.55	0.062	2.90 ±0.60	2.58 ±0.54	**0.014**
16. Apply health information to decided how treat disease	2.76 ±0.63	2.71 ±0.49	0.870	2.56 ±0.56	2.29 ±0.49	0.240	3.03 ±0.36	2.61 ±0.62	**0.002**	2.77 ±0.55	2.75 ±0.86	0.933	2.78 ±0.48	2.83 ±0.41	0.812	3.18 ±0.72	2.80 ±0.42	0.129	3.00 ±0.59	2.70 ±0.61	0.114	2.62 ±0.59	2.58 ±0.50	0.778
Total	11.16 ±1.89	10.57 ±1.51	0.431	10.67 ±1.76	9.43 ±1.99	0.102	12.00 ±1.10	10.61 ±2.20	**0.003**	11.32 ±2.00	11.00 ±3.43	0.721	11.32 ±1.31	11.17 ±1.60	0.792	12.96 ±2.59	10.80 ±1.55	**0.018**	12.17 ±2.01	10.81 ±2.13	**0.039**	10.90 ±1.96	10.23 ±1.76	0.109

Table S9. The association between age (<65y/o or ≥65 y/o) and **all scores** of MMHLQ divided by **PSGs** (Cont.)

	SLE			Head-and-neck cancer			DM			ADPKD			Hemodialysis			CKD			COPD			Osteoporosis		
	Age<65	Age≥ 65	*p* value	Age<65	Age≥ 65	*p* value	Age<65	Age≥ 65	*p* value	Age<65	Age≥ 65	*p* value	Age<65	Age≥ 65	*p* value	Age<65	Age≥ 65	*p* value	Age<65	Age≥ 65	*p* value	Age<65	Age≥ 65	*p* value
**Communication**																								
17. Talk to doctors the chosen examination and treatment	2.86 ±0.71	2.86 ±0.38	1.000	2.83 ±0.51	2.29 ±0.49	**0.012**	2.97 ±0.43	2.58 ±0.67	**0.007**	2.95 ±0.51	2.88 ±0.72	0.684	2.81 ±0.46	2.83 ±0.41	0.911	3.29 ±0.60	3.00 ±0.67	0.217	2.94 ±0.73	2.89 ±0.42	0.747	2.56 ±0.64	2.56 ±0.59	0.965
18. Make sure with medical personnel about accuracy of orders	2.98 ±0.48	2.86 ±0.38	0.520	2.78 ±0.72	2.71 ±0.49	0.825	2.97 ±0.36	2.71 ±0.59	**0.033**	2.94 ±0.56	2.75 ±0.68	0.249	2.95 ±0.47	2.83 ±0.41	0.582	3.32 ±0.55	3.00 ±0.47	0.093	3.00 ±0.59	2.89 ±0.42	0.467	2.79 ±0.57	2.67 ±0.61	0.358
19. Discuss with doctor about the choice of treatment	3.08 ±0.53	2.86 ±0.38	0.289	2.92 ±0.60	2.57 ±0.53	0.167	2.97 ±0.43	2.74 ±0.58	0.066	2.97 ±0.59	2.75 ±0.68	0.198	2.89 ±0.52	2.83 ±0.41	0.793	3.21 ±0.69	2.80 ±0.63	0.103	3.28 ±0.46	3.00 ±0.48	0.060	2.72 ±0.51	2.72 ±0.63	0.981
20. Ask medical personnel if you are not sure	3.00 ±0.35	2.86 ±0.38	0.326	2.89 ±0.57	2.57 ±0.53	0.184	3.00 ±0.40	2.71 ±0.64	**0.032**	3.00 ±0.56	2.81 ±0.66	0.249	2.97 ±0.44	2.83 ±0.41	0.471	3.29 ±0.60	3.00 ±0.47	0.143	3.22 ±0.55	2.93 ±0.55	0.083	2.82 ±0.56	2.67 ±0.61	0.260
Total	11.92 ±1.72	11.43 ±1.51	0.478	11.42 ±2.06	10.14 ±1.77	0.135	11.92 ±1.29	10.74 ±2.18	**0.010**	11.86 ±2.01	11.19 ±2.66	0.355	11.62 ±1.53	11.33 ±1.63	0.674	13.11 ±2.36	11.80 ±1.99	0.128	12.44 ±1.92	11.70 ±1.68	0.178	10.90 ±1.86	10.63 ±2.06	0.537

Table S10. The association between age (<65y/o or ≥65 y/o) and **all scores** of MMHLQ divided by **types** of PSGs

	Autoimmune disease			Malignancy			Chronic disease			Genetic disease			Degenerative disease		
	Age<65	Age≥65	*p* value	Age<65	Age≥65	*p* value	Age<65	Age≥65	*p* value	Age<65	Age≥65	*p* value	Age<65	Age≥65	*p* value
**Accessing**															
1.Searching information about disease	2.88 ±0.63	2.57 ±0.53	0.229	2.58 ±0.65	2.43 ±0.53	0.558	3.03 ±0.63	2.61 ±0.80	**0.022**	2.88 ±0.60	2.81 ±0.75	0.715	2.85 ±0.63	2.60 ±0.62	0.085
2.Get information about health protection	2.92 ±0.57	2.71 ±0.49	0.373	2.78 ±0.42	2.71 ±0.49	0.724	3.10 ±0.55	2.65 ±0.71	**0.005**	2.95 ±0.48	2.94 ±0.68	0.912	2.97 ±0.58	2.72 ±0.63	0.062
3.Find health information from network	3.10 ±0.62	2.71 ±0.49	0.120	2.81 ±0.62	2.71 ±0.49	0.717	3.00 ±0.73	2.45 ±0.81	**0.004**	3.00 ±0.53	2.69 ±0.95	0.220	3.05 ±0.51	2.58 ±0.82	**0.002**
4. Get information about report of health examination report	2.73 ±0.53	2.57 ±0.53	0.451	2.72 ±0.57	2.43 ±0.53	0.213	3.05 ±0.56	2.65 ±0.66	**0.008**	2.94 ±0.53	2.81 ±0.83	0.571	2.77 ±0.58	2.67 ±0.64	0.488
Total	11.63 ±1.84	10.57 ±1.40	0.150	10.89 ±1.89	10.29 ±1.70	0.439	12.18 ±2.21	10.35 ±2.70	**0.003**	11.77 ±1.91	11.25 ±3.07	0.526	11.64 ±1.90	10.58 ±2.24	**0.024**
**Understanding**															
5. Understand the instruction of medication bag	3.02 ±0.63	2.86 ±0.38	0.508	2.97 ±0.45	2.86 ±0.38	0.527	3.13 ±0.47	2.87 ±0.56	**0.041**	3.06 ±0.53	2.88 ±0.89	0.430	2.95 ±0.65	2.95 ±0.43	0.969
6. Obey the instruction of medical personnel to care disease	3.08 ±0.67	3.14 ±0.69	0.823	2.92 ±0.50	2.57 ±0.53	0.106	3.15 ±0.49	2.87 ±0.67	**0.045**	3.06 ±0.50	2.94 ±0.85	0.584	3.00 ±0.56	3.12 ±0.45	0.301
7. Understand the introduction of medical personnel	3.14 ±0.46	3.00 ±0.58	0.457	2.94 ±0.41	2.71 ±0.49	0.195	3.21 ±0.41	2.81 ±0.65	**0.003**	3.09 ±0.46	2.88 ±0.72	0.264	3.08 ±0.53	3.07 ±0.46	0.948
8. Follow the instruction of medical bag to take medication	3.10 ±0.59	3.00 ±0.58	0.665	3.08 ±0.37	3.00 ±0.00	0.557	3.15 ±0.49	2.94 ±0.68	0.123	3.12 ±0.55	2.94 ±0.68	0.249	3.10 ±0.60	3.12 ±0.59	0.917
Total	12.41 ±1.91	12.00 ±2.08	0.603	11.92 ±1.20	11.14 ±1.07	0.122	12.64 ±1.60	11.48 ±2.29	**0.015**	12.34 ±1.81	11.63 ±2.92	0.362	12.13 ±1.88	12.26 ±1.51	0.735

Table S10. The association between age (<65y/o or ≥65 y/o) and **all scores** of MMHLQ divided by **types** of PSGs (Cont.)

	Autoimmune disease			Malignancy			Chronic disease			Genetic disease			Degenerative disease		
	Age<65	Age≥ 65	*p* value	Age<65	Age≥65	*p* value	Age<65	Age≥65	*p* value	Age<65	Age≥65	*p* value	Age<65	Age≥ 65	*p* value
**Evaluation**															
9. Evaluate whether the health information can be used to solve medical problems	2.69 ±0.58	2.86 ±0.38	0.346	2.64 ±0.49	2.29 ±0.49	0.087	2.95 ±0.56	2.58 ±0.62	**0.012**	2.72 ±0.65	2.69 ±0.79	0.852	2.62 ±0.63	2.47 ±0.63	0.285
10. Evaluate the health information suitable for himself/herself or not	2.65 ±0.63	2.71 ±0.49	0.807	2.64 ±0.54	2.43 ±0.53	0.353	2.90 ±0.60	2.55 ±0.68	**0.025**	2.78 ±0.57	2.75 ±0.77	0.841	2.59 ±0.59	2.53 ±0.59	0.677
11. Evaluate the difference or consistence of health information	2.45 ±0.65	2.43 ±0.53	0.937	2.69 ±0.52	2.43 ±0.53	0.228	2.82 ±0.60	2.55 ±0.77	0.111	2.75 ±0.64	2.75 ±0.86	0.984	2.49 ±0.68	2.44 ±0.55	0.740
12. Evaluate the reliability of medical information from network	2.51 ±0.68	2.29 ±0.49	0.405	2.58 ±0.65	2.71 ±0.49	0.616	2.62 ±0.71	2.35 ±0.75	0.143	2.68 ±0.64	2.75 ±0.86	0.704	2.38 ±0.71	2.21 ±0.60	0.230
Total	10.31 ±2.11	10.29 ±1.50	0.975	10.56 ±1.87	9.86 ±1.68	0.365	11.28 ±2.18	10.03 ±2.51	**0.029**	10.94 ±2.19	10.94 ±3.21	0.999	10.08 ±2.25	9.65 ±1.90	0.356
**Application**															
13. Apply health information to know the progress of disease	2.71 ±0.61	2.43 ±0.53	0.247	2.69 ±0.58	2.29 ±0.76	0.110	2.97 ±0.43	2.61 ±0.62	**0.008**	2.78 ±0.57	2.75 ±0.86	0.880	2.62 ±0.54	2.51 ±0.51	0.373
14. Apply health information to prevent disease	2.84 ±0.55	2.71 ±0.49	0.581	2.69 ±0.47	2.29 ±0.76	0.210	2.97 ±0.36	2.65 ±0.66	**0.017**	2.85 ±0.57	2.75 ±0.86	0.674	2.77 ±0.54	2.56 ±0.59	0.095
15. Apply health information to understand the report of health examination	2.86 ±0.58	2.71 ±0.49	0.536	2.72 ±0.57	2.57 ±0.53	0.519	3.03 ±0.28	2.74 ±0.58	**0.016**	2.92 ±0.51	2.75 ±0.86	0.448	2.90 ±0.60	2.58 ±0.54	**0.014**
16. Apply health information to decided how treat disease	2.76 ±0.63	2.71 ±0.49	0.870	2.56 ±0.56	2.29 ±0.49	0.240	3.03 ±0.36	2.61 ±0.62	**0.002**	2.77 ±0.55	2.75 ±0.86	0.933	2.62 ±0.59	2.58 ±0.50	0.778
Total	11.16 ±1.89	10.57 ±1.51	0.431	10.67 ±1.76	9.43 ±1.99	0.102	12.00 ±1.10	10.61 ±2.20	**0.003**	11.32 ±2.00	11.00 ±3.43	0.721	10.90 ±1.96	10.23 ±1.76	0.109

Table S10. The association between age (<65y/o or ≥65 y/o) and **all scores** of MMHLQ divided by **types** of PSGs (Cont.)

	Autoimmune disease			Malignancy			Chronic disease			Genetic disease			Degenerative disease		
	Age<65	Age≥ 65	*p* value	Age<65	Age≥65	*p* value	Age<65	Age≥65	*p* value	Age<65	Age≥65	*p* value	Age<65	Age≥ 65	*p* value
**Communication**															
17. Talk to doctors the chosen examination and treatment	2.86 ±0.71	2.86 ±0.38	1.000	2.83 ±0.51	2.29 ±0.49	**0.012**	2.97 ±0.43	2.58 ±0.67	**0.007**	2.95 ±0.51	2.88 ±0.72	0.684	2.56 ±0.64	2.56 ±0.59	0.965
18. Make sure with medical personnel about accuracy of orders	2.98 ±0.48	2.86 ±0.38	0.520	2.78 ±0.72	2.71 ±0.49	0.825	2.97 ±0.36	2.71 ±0.59	**0.033**	2.94 ±0.56	2.75 ±0.68	0.249	2.79 ±0.57	2.67 ±0.61	0.358
19. Discuss with doctor about the choice of treatment	3.08 ±0.53	2.86 ±0.38	0.289	2.92 ±0.60	2.57 ±0.53	0.167	2.97 ±0.43	2.74 ±0.58	0.066	2.97 ±0.59	2.75 ±0.68	0.198	2.72 ±0.51	2.72 ±0.63	0.981
20. Ask medical personnel if you are not sure	3.00 ±0.35	2.86 ±0.38	0.326	2.89 ±0.57	2.57 ±0.53	0.184	3.00 ±0.40	2.71 ±0.64	**0.032**	3.00 ±0.56	2.81 ±0.66	0.249	2.82 ±0.56	2.67 ±0.61	0.260
Total	11.92 ±1.72	11.43 ±1.51	0.478	11.42 ±2.06	10.14 ±1.77	0.135	11.92 ±1.29	10.74 ±2.18	**0.010**	11.86 ±2.01	11.19 ±2.66	0.355	10.90 ±1.86	10.63 ±2.06	0.537
